# From Arksey and O’Malley and Beyond: Customizations to enhance a team-based, mixed approach to scoping review methodology

**DOI:** 10.1016/j.mex.2021.101375

**Published:** 2021-05-07

**Authors:** Kristi K Westphaln, Wendy Regoeczi, Marie Masotya, Bridget Vazquez-Westphaln, Kaitlin Lounsbury, Lolita McDavid, HaeNim Lee, Jennifer Johnson, Sarah D. Ronis

**Affiliations:** a Department of Bioethics, Case Western Reserve University School of Medicine, Cleveland, OH, USA; b UH Rainbow Babies and Children’s Hospital, Cleveland, OH, USA; c College of Liberal Arts and Social Sciences, Cleveland State University, Cleveland, OH, USA; d UH Rainbow Center for Child Health and Policy, Cleveland, OH, USA; e Memorial Care, Miller Children and Women’s Hospital, Long Beach, CA, USA; f National Children’s Alliance, Washington, DC, USA; g Department of Pediatrics, Case Western Reserve University School of Medicine, Cleveland, OH, USA; h Department of Social Welfare and Counseling, Dongguk University, South Korea; i Canopy Children’s Advocacy Center, Cleveland, OH, USA

**Keywords:** Scoping review, REDCap, Mixed method, Team-based, Children’s advocacy centers, CA

## Abstract

This article presents a method of conducting a scoping review that synthesizes recommendations from previous literature while contributing additional customizations to enhance a team-based, mixed method approach. A form of knowledge synthesis, scoping reviews aim to describe the breadth of an existent knowledge base and inform future research, practice, and policy. Scoping review methodology has continued to evolve since the inception of the Arksey and O’Malley Framework in 2005. After examining recommendations from the body of literature on scoping review methodology and scoping reviews about scoping reviews, we found that teamwork and mixed method approaches were rarely addressed. Following the Arksey and O’Malley Framework, we discuss current recommendations within the literature, rationale for our customizations to enhance the method, and present an application of these customizations as illustrated in our companion article, “Outcomes and outputs affiliated with children’s advocacy centers in the United States: A scoping review.”In sum, our enhancements to the Arksey and O’Malley Framework:

• Explicitly integrate qualitative and quantitative assessment of the literature following best practices in mixed methods research, and,
• Integrate a team-based approach throughout all stages of the scoping review process.

## Graphical Abstract

**Figure:**
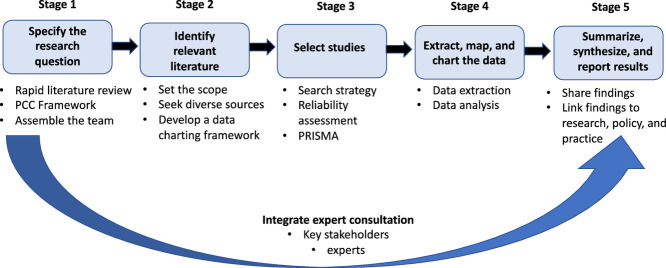

Specifications table

**Table:** 

Subject Area:	Medicine and Dentistry
More specific subject area:	Outcome and Output Measures affiliated with Children’s Advocacy Centers, Child Abuse
Method name:	*Scoping review*
Name and reference of original method:	Arksey, H & O’Malley, L. (2005). Scoping Studies: Towards a methodological framework. *International Journal of Social Research Methodology*. 8(1):19-32.
Resource availability:	REDCap: https://www.project-redcap.org

## Method overview

### Rationale

Scoping reviews are a method of knowledge synthesis that identify trends and gaps within an existent knowledge base, or scope of knowledge, for the purpose of informing research, policy, and practice [2]; Levac [11]; Tricco et al. [15]. Since the debut of Arksey and O’Malley's seminal article on scoping review methodology [2], the method continues to evolve. The purpose of this manuscript is to summarize, synthesize, and offer customizations to enhance scoping review methodology within the context of a mixed method, team-based approach.

Arksey and O’Malley [2] were the first to highlight the rationale and provide methodological recommendations for conducting a scoping review. They articulate four common reasons to undertake a scoping review: 1) examine the state of research activity on a selected topic, 2) screen for the need for and/or feasibility of undertaking a systematic review, 3) synthesize and share research findings, and/or 4) highlight key gaps in the literature [2]. To assist authors through the scoping review process, the Arksey and O’Malley Framework [2] propose six stages of conduct: 1) specify the research question, 2) identify relevant literature, 3) select studies, 4) map out the data, 5) summarize, synthesize, and report the results, and 6) include expert consultation.

Scholars continue to contribute meaningful insights to build upon the Arksey and O’Malley Framework. Levac et al [11] were the first to call attention to the need for universal language to consistently describe the method. Given the inconsistent use of nomenclature such as “scoping review,” “scoping exercise,” “scoping study,” or “scoping review of the literature” in the literature, scholars continue to advocate for the use of “scoping review” as a universal term of reference [4], [11], [12], [14]. Ambiguity remains a main critique of the initial Arksey and O’Malley framework. Levac et al [11] address this by offering detailed descriptions and the challenges associated with each stage of the Arksey and O’Malley framework. Further methodological recommendations on the conduct of scoping reviews are published by Colquhoun et al [4], Daudt, van Mossel, and Scott [6] and the Joanna Briggs Institute in [13]. Colquhoun et al [4] call to improve transparency in reporting and suggest using an adaptation of the Preferred Reporting Items for Systematic Reviews and Meta-Analyses (PRISMA) and conversations continue towards constructing a standardized reporting checklist via the Enhancing the QUAlity and Transparency of Health Research (EQUATOR) [15]. By comparing and contrasting scoping and systematic reviews, Joanna Briggs Institute [13] clarified the purpose and essential characteristics associated with each type of review and essential elements to include when creating a protocol for a scoping review.

As knowledge and use of scoping review methodology increased, so have the number of published scoping review manuscripts [15]. A PubMed search of the term “scoping review” yielded 27,345 results as of October 23, 2020 and revealed a trend of scoping reviews on scoping reviews [1], [7], [14], [15]. Findings synthesized from the scoping reviews on scoping reviews revealed critiques including wide variation in methodological rigor [1], [14],15]; inconsistencies in how findings are reported [7,^14^,15]; lack of reference to quality [14,15]; and limited guidance on team-based applications of the method [6,11]. Furthermore, the inclusion of quality appraisal or risk of bias remains under debate [2], [6]; Levac et al, 2014; [4,^13^,14], as does the question of whether scoping reviews as a methodology would benefit from the construction and application of standardized reporting guidelines [4,^13^,^14^,15].

**Method details: Enhancing the Arksey and O’Malley Framework**

This paper builds upon the existent literature describing scoping review methodology, with customizations that emphasize a team-based, mixed method approach. Our recommended enhancements, as derived from our synthesis of literature on scoping review methodology and findings from scoping reviews about scoping reviews, are organized into tables per each stage of the Arksey and O’Malley Framework. We present our rationale for these methodological customizations, illustrating how we applied these customizations within “Outcomes and outputs affiliated with children’s advocacy centers in the United States: A scoping review” (2020).

**1) Arksey and O’Malley Framework Stage 1: Specify the research question**

Clear articulation of the research question is the first stage of conducting a scoping review [2]. While the main challenges associated with this stage involve the broad nature of the research question(s) and purpose [Bibr bib0004][Bibr bib0011], key strategies identified in prior literature for success in this stage include: embracing the process of question specification for scoping review as iterative, refining the question, clarifying its relationship to the study purpose, and determining the relevant population and context in conversation with learning from existent literature (Table 1A). To these recommendations, we add “identify framework within which to refine specification of research question and purpose.” (Table 1B).

**Table 1A:** Stage 1: review of the literature.

Stage1: Specify the research question	Source
- Identify the research question and consider how the question informs the search strategy - Maintain a broad approach - Refrain from setting specific parameters for the search strategy without an understanding of the scope of the existent literature - Consider the process iterative	[2]
- Clearly state the research question, purpose, and how they relate to each other - Specify the population, concepts, and context	[11]
- Link the research question and purpose - Frame refining the research question and search terms as an iterative process	[6]
- Specify population, concept, and context	[13]

**Table 1B:** Stage 1: recommended enhancements.

Stage 1	Recommended enhancements
Specify the research question	- Rapid review to scan the scope of the existent literature - Specify population, concepts, and context - Cleary state the research question(s) and purpose - Use cues from the literature to assemble a research team with the appropriate knowledge and expertise - Identify authors with expertise in the field of interest - Keep the process iterative - Set a time line and communication plan with team members

**Research question and purpose: “Outcomes and outputs affiliated with children’s advocacy centers in the United States: A scoping review”**

We first conceptualized our research questions and purpose based on a rapid review of the literature. This broad search revealed a few important insights. While a systematic review exploring the efficacy of the Children’s Advocacy Center (CAC) model was published in 2016 [10], the results of the systematic review were limited because very few articles were able to meet the rigorous inclusion criteria required of a systematic review. Additionally, the rapid review of the literature suggested that a wide variety of disciplines publish articles about CACs. This is not an uncommon trend within research about child abuse and neglect, as caring for children and families who experience abuse often involves multiple sectors, disciplines, and professionals. Given this, it became clear that a team-based approach (the term team-based approach will be used in this manuscript to include all types of teams: multidisciplinary, interdisciplinary, transdisciplinary, and interprofessional) would be necessary in order to best capture diverse perspectives within the literature. The rapid review also revealed a need to clearly articulate the definitions of key concepts for the scoping review as different disciplines define outputs and outcomes in different ways.

We selected a population, concepts, and context (PCC) framework to refine the research question and purpose.

**Population:** Children aged 0-18 years old who experienced child abuse, non-offending family members of children who experienced child abuse, and multidisciplinary team members who serve children and families who experience child abuse (law enforcement, prosecution, medial team, mental health, victim advocates, CAC staff, social work) in the United States.

**Concept:** 1. Outcome and output measures affiliated with CACs

2. Outcome measures were defined as person-centered

3. Output measures were defined as programmatic or service characteristics

**Context:** Must involve CACs

The research questions were:

• What types of person-oriented outcomes are being measured among children and non-offending family members who seek services and resources at CACs in the United States?
• What types of output measures are affiliated with CACs in the United States?
• What types of research designs are being used to study CACs?

We articulated our research purpose as follows:

A greater understanding of the scholarly work surrounding outcome and output measures used by CACs will identify gaps in the research, clarify key themes/concepts, and highlight opportunities to enhance outcomes for children and families who experience abuse and receive services via CACs.

**2) Arksey and O'Malley Framework Stage 2: Identify relevant literature**

The second stage of the Arksey and O’Malley Framework focuses on identifying relevant literature, as guided by the research question and purpose [2]. While practical issues such as timing and resources are cited as barriers that may limit the scope of the literature [11], Table 2A depicts recommendations to navigate such challenges.

**Table 2A:** Stage 2: identify relevant literature.

Stage 2: Identify relevant literature	Source
- Be comprehensive: include published, unpublished, or review manuscripts - Specify decisions about scope, such as time frame or language - Helpful sources for information: internet, electronic databases, article reference lists, hand searching, networking at conferences, or relevant professional organizations	[2]
- Assemble a team with appropriate knowledge and expertise - Use the research question and purpose to guide the scope of the study - Clearly acknowledge and justify decisions that limit the scope of the study	[11]
- Additional helpful sources of information such as dissertations, closed clinical trials, clinical practice guidelines, or the Grey Matters Checklist - Consider a small team vs a large team to be involved in the research process - Invite a librarian to join the team to guide the search strategy - Online citation management software can be very helpful	[6]
- Create an a priori scoping review protocol	[13]
- Consider personal communication as part of the search strategy	[15]

**A team-based approach in scoping review methodology**

While Arksey and O’Malley [2] broadly addressed the importance of integrating team-based approach in scoping review methodology, Levac et al [11] specifically called for the explicit incorporation of teams into the method as a means to provide content expertise. As an example, Daubt et al [6] applied the Arksey and O’Malley Framework within the context of an inter-professional team, which they defined as including members with both clinical and research training. Some of the identified challenges of implementing a team-based approach w within the scoping review process include decreased productivity by increasing challenges in coordinating with multiple members, reaching consensus, and navigating a lack of common language [6]. Despite these challenges, the rich insights from diverse perspectives will meaningfully enhance the scoping review process and findings [2,^6^,11]. Daubt et al [6] suggest that regular consistent communication, regular meetings, designated team leads, and a smaller research team help to facilitate successful inter-professional teamwork for scoping reviews.

**Assembling the research team for “Outcomes and outputs affiliated with children’s advocacy centers in the United States: A scoping review”**

Data from the rapid review in stage 1 clearly identified the need for a team-based approach in answering the proposed research questions. Rather than providing a specific name for the type of team, a framework was developed to clearly emphasize the var various roles, expertise, skills, and contexts embodied within the members of our team. Given the diverse systems that respond to child abuse and neglect, the research team was selected to include representation from nursing (KKW), criminology (WR), public health (MM, KL), clinical psychology (BVW), medicine (SDR, LM), and social work (HL). Most of the members of the research team possessed both research and clinical expertise. Two team members were selected for their content expertise specifically related to CACs (KL, JJ).

**Research:** Four of the team members possess doctoral degrees (KKW, HL, WR, BVW), one of the members is a doctoral candidate (SDR), and the two team members with public health degrees are employed in research positions (MM, KL).

**Clinical Practice:** A general pediatrician (SDR), a board-certified child abuse pediatrician (LM), a pediatric nurse practitioner with experience as a sexual assault forensic investigator and working on children protection teams (KKW), a child and family psychologist (BVW), and a criminologist with expertise in collaborating with law enforcement (WR).

**Policy:** KL is affiliated with the National Children’s Alliance (NCA), the national accreditation body for CACs. JJ is the Director of the Canopy Child Advocacy Center in Cleveland, Ohio.

Descriptive analysis of our sample revealed that the most predominant disciplinary backgrounds of the first authors included psychology (29%), social work (22%), and nursing (11%) (Supplementary Material A); thereby validating the importance of having a psychologist, social worker, and nurse as members of the research team.

**Search Strategy and Abstract Selection: “Outcomes and outputs affiliated with children's advocacy centers in the United States: A scoping review”**

In keeping with the Arksey and O’Malley Framework goal of comprehensiveness, we developed our search strategy to be inclusive of publications within the full time frame applicable to our research question (1985 to 2019), relevant to the setting of interest (U.S.), and population of interest (children). We searched multiple indexing services that addressed disciplines relevant to our research question using the key terms of “Child Advocacy Center(s),” “Children’s Advocacy Center(s),” and “CAC(s)”, including: CINAHL (KKW), PubMed (KKW, AG), SocIndex (WR), Ovid (KKW), and PsychINFO (KKW). The search was extended to include a backwards review of citations from key peer reviewed and grey literature (KKW), hand search (KKW), and recommendations from expert consultants (JH, TC, and WW).

**Article Storage and Data Collection: “Outcomes and outputs affiliated with children’s advocacy centers in the United States: A scoping review”**

While recommendations to enhance stage 2 of the Arksey and O’Malley Framework focus on identifying the relevant literature to include in the scoping review, sparse details are provided about how to store and organize the articles. Full text article review and data extraction/collection can easily become challenging when scoping reviews involve multiple articles and reviewers. Prior to selecting the studies for stage 3, it may be helpful to establish a plan for article storage and data collection (Table 2B).

**Table 2B:** Stage 2: recommended enhancements.

Stage 2	Recommended enhancements
Identify relevant literature	- Describe rationale for decisions that limit the scope, such as language or time frame - Invite a librarian to help guide the search - Seek diverse published and unpublished sources: internet, electronic databases, article reference lists, hand searching, networking at conferences, relevant professional organizations, dissertations, closed clinical trials, clinical practice guidelines, or the Grey Matters Checklist - Use a research, clinical practice, policy framework to identify the knowledge and expertise of team members - Identify and reach out to potential expert consultants - Create an a priori scoping review protocol - Consider using online management software such as REDCap - Train team members on how to use the online management software - Determine the variables of interest for data extraction per the research question - Discuss variable candidates with the research team - Develop a data charting framework: consider incorporating quantitative and qualitative Components

We used Research Electronic Data Capture software (REDCap) to store articles and facilitate data extraction. REDCap is a secure, web-based platform that supports data capture for research [8], [9]. REDCap supports the inclusion of multiple users within projects, accommodates multiple types of data, provides audit trails for tracking data manipulation and exportation, and facilitates data exportation into many types of statistical software [8], [9]. These functions allow REDCap to efficiently accommodate the process of full text review and data extraction for large research teams (Table 2B).

**3) Arksey and O’Malley Framework Stage 3: Select studies**

The third stage of the Arksey and O’Malley Framework refers to the process of selecting studies for the scoping review. Different than the linear format of systematic reviews, the study selection process for a scoping review is iterative and therefore more challenging to clearly capture [11]. Table 3A describes recommendations from the literature to enhance transparency and reproducibility of study selection for scoping reviews.

**Table 3A:** Stage 3: select studies.

Stage 3: Select studies	Source
- Define the inclusion and exclusion criteria after understanding of the scope of the literature - Consider using two independent reviewers to apply inclusion and exclusion criteria	[2]
- Consider the search strategy as an iterative process of reviewing the literature, revising the strategy, and eligibility for inclusion - Specify inclusion and exclusion criteria as guided by the research question and purpose - At least two reviewers should independently review abstracts and full text articles for inclusion - Assign a third reviewer to address inquiries or disagreements regarding inclusion	[11]
- Pilot a three-tiered approach to engage team members in the study selection process: 1. divided the team into 6 teams of 2 people, assign each team 1/6^th^ of the articles to review, team members independently review of full text articles 2. team members compared results regarding exclusion or inclusion 3. a third team member resolved any discrepancies	[6]
- At least 2 reviewers for inclusion/exclusion - Follow the Joanna Briggs Institute guidelines for systematic reviews: 1. Initial search of relevant databases, article title and abstract review 2. Search key terms 3. Search reference lists from all identified articles - Include the Preferred Reporting Items for Systematic Reviews and Meta-Analyses (PRISMA) flow chart	[13]
- Two groups of reviewers: group 1 for title and abstract search, group 2 for full text inclusion and exclusion - Resolve discrepancies regarding inclusion by a single arbitrator - Consider calculating inter-rater agreement during the study selection process	[15]

**Full text review: “Outcomes and outputs affiliated with children’s advocacy centers in the United States: A scoping review”**

Once the final sample of articles eligible for full text analysis was determined, articles were assigned by the lead author (KKW) to match with the disciplinary, research, clinical, or policy expertise of each reviewer. This was done purposefully to maximize the diverse strengths of our review team. Each team member screened their assigned articles per the inclusion criteria for full text review: the article must involve CACs, feature child victims less than 18 years of age, take place in the United States, and be written in English. Discrepancies involving article selection were discussed with the lead author (KKW) until consensus was achieved to include or exclude the article in the full text analysis. The search strategy and article selection process were reported using a PRISMA flowchart. The PRISMA flowchart is accessible as Figure 1 in our companion article, “Outcomes and outputs affiliated with children’s advocacy centers in the United States: A scoping review” (Table 3B).

**Table 3B:** Stage 3: recommended enhancements.

Stage 3	Recommended enhancements
Select Studies	- Clearly define inclusion and exclusion criteria for title and abstract search and full text search - Assign a unique identifying number to each article to be included in the full text review - Assign at least 2 team members to independently review titles and abstracts for inclusion - Specify the team member who will address any questions that arise with the selection of titles and abstracts - Assign 2 different team members to independently conduct a full text review for each article, and a third person to address questions or resolve discrepancies involving inclusion or exclusion - Consider assigning team members to review articles that match their knowledge and expertise - Integrate some aspect of interrater reliability assessment, such interrater agreement or a pooled kappa, during the study selection process - Use the Preferred Reporting Items for Systematic Reviews and Meta-Analyses Flowchart (PRISMA) to enhance reporting transparency

**Variables for Data Extraction Framework: “Outcomes and outputs affiliated with children’s advocacy centers in the United States: A scoping review”**

Data extraction fields and variable selection selectoion were informed by the Arksey and O’Malley Framework (2005), discussion with expert contributors, and research by Peters et al [13], Herbert and Bromfield [10], and Cross et al. (2008). Variables were identified a priori to capture both the characteristics and the content of the manuscripts selected for inclusion in full text review. Manuscript characteristic variables included: title, journal, author, discipline of lead author, year of publication, and funding status. Manuscript content variables addressed the domains of: study purpose/aims, research design, population, setting, outcomes, outputs, findings, and future recommendations (Supplementary Material B).

We defined outcome measures for CACs as those measures that are person-centered, meaning involving a person and the subject for the outcome. For each article included in full text review, we first assessed whether or not person-centered outcomes were addressed. If yes, we then documented a conceptual description of the outcome, the operational definition for the outcome, the subject of the outcome (child, family member, family unit, CAC staff or MDT member, other), and the type of outcome (mental health, physical health, forensic interview, disclosure of abuse, professional practice, knowledge, prosecution, attitudes, policy, satisfaction, other (Supplementary Material C).

Distinct from person-centered outcomes, we defined output measures as those as findings involving programmatic or service characteristics that demonstrate functionality of the CAC model. For each article included in full text review, we assessed whether any CAC-relevant outputs were reported. If yes, we documented the conceptual definition of the output, the operational definition of the output, and the service line associated with the output (CAC staff, prosecution, forensic interview, law enforcement, economic, medical services, victim advocates, mental health, child protection services, combination, or other (Supplementary Material D).

**4) Arksey and O’Malley Framework Stage 4: Extracting, mapping, and charting the data**

Stage four of the Arksey and O’Malley [2] Framework refers to the use of a type of descriptive analytical method to conduct data charting or mapping. While this process is known as data extraction within the context of a systematic review or would be defined per specific statistical techniques within the context of a meta-analysis, a standard process of mapping or charting the data for a scoping review has not been clearly delineated. Arksey and O’Malley [2] describe data charting/mapping as a technique to synthesize and interpret qualitative data, however the steps to replicate this technique are not articulated in the Framework [11]. Additionally, navigating how much data to include or not include can be challenging, as can the question of how to integrate numeric data with qualitative findings. Table 4A presents a synthesis of current recommendations for stage four of the Arksey and O’Malley Framework.

**Table 4A:** Stage 4: extracting, mapping, and charting the data.

Stage 4: Mapping/Extracting/Charting the data	Source
- “Charting” the data is defined as the process of synthesizing and interpreting qualitative data according to themes - “descriptive-analytical” method is defined as the application of a common analytical framework	[2]
- Determine the variables of interest for data extraction per the research question - Discuss variable candidates with the research team - Develop a data charting framework - The data extraction and charting process should first be piloted by at least two members of the research team prior to conducting the entire review - Consider the nature of the variables of interest when developing the data analysis plan. - Recommend consideration of qualitative content analysis approach to gain a deeper understanding of the data	[11]
- Adapted a framework from a systematic review that was a good match to the research question - The process of engaging with and capturing the information of interest was perceived as challenging by some of the team members - Implementing a trial charting exercise followed by group discussion improved the quality and consistency of the charting/mapping - Conducted an additional three-tiered process, similar to in stage 3, for the charting procedure. - Inter-reviewer reliability: A member of the research team read and charted each article, then compared this to the findings from the teams - Communication is key for conducting scoping reviews in large teams - Assignment of a unique identifying number to each article to be included in the full text review	[6]
- Present findings as a map of outcomes - Discussion section should emphasize the findings within the context of the current trends in the literature and implications for policy and practice	[13]
- Quantitative analysis - Qualitative analysis: two team members conducted initial categorization, coding, and charting of relevant texts - Developed a framework for analysis via team discussion of preliminary results - Word cloud application	[15]

**A mixed methods approach applied to scoping review methodology**

As defined by Creswell and Plano-Clark (2018), mixed method research combines methods, designs, and philosophical orientation to: 1) collect and analyze quantitative and qualitative data to answer a research question or explore a hypothesis, 2) integrate the two types of data, 3) utilize specific research designs to conduct the research, and 4) ground the research within philosophy and theory. Researchers often employ mixed method approaches to explore and corroborate results, obtain a comprehensive understanding of context prior to conducting a study, describe and compare data from multiple sources, or enhance one method by using another, such as conducting a qualitative study to better understand the results from a quantitative study [5]. Given this, a mixed method approach is well aligned with the broad purpose of scoping reviews. Previous scholars [6,^11^,15] address using content and numerical analyses in scoping reviews, however this has not yet been formally referred to as a mixed method approach, nor incorporated as a stage within the Arksey and O’Malley Framework (2005). Some of the advantages of incorporating a mixed approach (quantitative and qualitative) into scoping review methodology are listed below in Table 4B.

**Table 4B:** Advantages: incorporating a mixed approach to scoping review methodology adapted from [5]

**Strengths of a Mixed Method Approach:** - Answer a variety of research questions - Harness the strengths while offsetting the weaknesses of using one solitary method - Provide more evidence - Encourage diverse perspectives (multiple stakeholders, disciplines, professions, worldviews) - Enhance opportunities for collaboration - Solve problems with both numbers and words

**Table 4C:** Stage 4: recommended enhancements.

Stage 4	Recommended enhancements
Extracting, mapping, and charting the data	- Pilot the data extraction process with at least 2 team members prior to launching it across the entire team - Data analysis: quantitative (numerical analysis), qualitative (specify qualitative method, such as content analysis) - Consider side by side comparison of quantitative and qualitative data

**Assessment of Interrater reliability in scoping review methodology**

Interrater reliability represents consistency or agreement among raters or reviewers [16]. Often expressed as a correlation coefficient such as a kappa or pooled kappa, it can also be expressed as a percentage of agreement. While Daubt et al [6] discuss a customized three-tiered approach to the study selection process, Tricco et al [15] are the first to recommend including an assessment of interrater reliability as a means to enhance the validity of the scoping review process.

**Extracting, mapping, and charting the data: “Outcomes and outputs affiliated with children’s advocacy centers in the United States: A scoping review”**

A pilot of the full text data extraction process was conducted to assess the feasibility and acceptability of using the REDCap database. A few minor modifications were made to the REDCap database to enhance usability (drop down menus and other comment boxes) and further clarify the definitions of study variables (implementation science/research, outputs, and outcomes). Reviewers independently performed data extraction from their individually assigned articles that met the inclusion criteria and entered it into the REDCap database. Similar to the process for article selection, reviewer inquiries about data fields or data extraction were discussed with either KKW or MM. Reviewer feedback was repeatedly assessed and utilized to inform improvements in the structure and use of the REDCap database in this review.

**Interrater Proportion Agreement: “Outcomes and outputs affiliated with children’s advocacy centers in the United States: A scoping review”**

Interrater proportion agreement was assessed to determine the consistency among reviewer data entry into REDCap. A total of 10 articles were randomly selected from the sample of articles that underwent full text analysis. Each of the randomly selected articles underwent an additional full text analysis, data extraction, and data entry process by the lead author of this paper (KKW). Consistency of data entry into REDCap was compared between the original reviewer and secondary reviewer, with agreement status measured by yes or no. The interrater proportion agreement was 83%. (Supplementary Material E).

**5) Arksey and O’Malley Framework Stage 5: Summarize, synthesize, and report the results**

Stage five of the Arksey and O’Malley Framework [2] refers to the process of constructing an analytic framework that describes the breadth of the literature and identifies priority areas within that literature. Given that there are multiple steps involved in the collating, synthesizing, and reporting process, the main challenge of stage 5 is that it lacks clear guidance on how to systematically accomplish this.

Levac et al [11] build upon the Arksey and O’Malley Framework by suggesting three distinct steps for stage five: 1) conduct quantitative descriptive analysis and qualitative thematic analysis, 2) report the results within the context of the research question(s) and purpose, and 3) interpret the findings within the context of future research, practice, and policy. These recommendations in Table 5A address the benefit of including both quantitative and qualitative data within scoping reviews, and support the consideration of integrating a mixed method framework into scoping review methodology. Table 5B highlights additional customizations.

**Table 5A:** Stage 5: Summarize, synthesize, and report the results.

Stage 5: Summarize, synthesize, and report the results	Source
- Develop a framework or template to summarize and analyze the results - Numerical analysis	[2]
- Analyze the data (quantitative or qualitative) - Relate the results to the research question and/or purpose. - Present findings within the context of future research, policy, and practice.	[11]
- Search for a large range of data to meet the interests of the inter-disciplinary team members - Provide additional level of depth to the data to have expertise from multiple disciplines - Prioritize findings to emphasize implications for future research - Quantitative analysis and thematic analysis	[6]
- Apply PRISMA	[13]

**Table 5B:** Stage 5: Recommended Enhancements.

Stage 5	Recommended enhancements
Summarize, synthesize, and report the results	- Present findings (charts, tables, maps, word clouds) - Relate the findings to the research question(s) and purpose - Discussion section should apply the findings within the context of current trends and future implications for research, practice, and policy.

**Data Analysis: “Outcomes and outputs affiliated with children’s advocacy centers in the United States: A scoping review”**

Data were exported from REDCap into a Microsoft Excel spreadsheet, inspected (KKW), cleaned (KKW), and separated into quantitative and qualitative databases prior to analysis (KKW). Guided by our convergent mixed method design, the qualitative and quantitative data were analyzed separately. For the quantitative strand, we used SPSS software version 26 to calculate frequencies and percentages. For the qualitative strand, we applied Braun and Clarke’s [3] method of thematic analysis to identify and analyze patterns in our qualitative data. The qualitative data were independently sorted and coded by two members of the research team (KKW, WR), with a third team member serving as a thematic auditor (BVW). Preliminary themes were discussed with the research team and then grouped into thematic maps. Findings from each strand were integrated and discussed within the context of trends, gaps, and implications for research, practice, and policy. (Supplementary Materials F and G).

**6) Arksey and O’Malley Framework Stage 6: Integrate expert consultation**

The importance of integrating expert consultation within the scoping review process is consistently emphasized within the literature describing scoping review methods and scoping reviews of scoping reviews [2,^4^,^6^,^11^,15]. Many scholars agree that inviting expert consultation into the process provides key feedback for scoping reviews and integrates knowledge that may not be accessible via academic literature, such as cutting-edge trends that may not yet be published and valuable historical context. However, recommendations vary in the literature on the timing and extent of engaging with expert consultants across the scoping review process [2,11]. Additionally, the initial Arksey and O'Malley Framework (2005) presents expert consultation as an optional component, whereas Levac et al [11] consider this to be an essential component for scoping review methodology. Table 6A presents a synthesis of the literature for stage 6 of the Arksey and O’Malley Framework.

**Table 6A:** Arksey and O’Malley Framework Stage 6: Integrate expert consultation.

Stage 6: Integrate expert consultation	Sources
- Incorporates knowledge from experts and other key stakeholders - Consultation offers added contextual value - Useful in gathering valuable insights that may not be visible within the literature	[2]
- Plan to engage in knowledge exchange with expert stakeholders in the field of interest - Articulate rationale for expert consultation - Specify degree of involvement for expert consultants - Enhance the validity of the study	[11]
- View consultation as an essential to the method - Consider consultant participation throughout the scoping review	[6]
- Define consultation as a knowledge translation activity	[15]

**Expert consultation: “Outcomes and outputs affiliated with children’s advocacy centers in the United States: A scoping review”**

The rapid review of the literature from the first stage of the Arksey and O’Malley Framework identified leading authors in the field of research on Children’s Advocacy Centers. Three experts were selected and consulted early and throughout the scoping review process. All three experts (JH, WW, TC) kindly offered valuable personal insights as well as additional relevant literature to add to the search that positively impacted the trajectory of the scoping review (Table 6B).

**Table 6B:** Stage 6: Recommended Enhancements.

Stage 6	Recommended enhancements
Integrate expert consultation	- Incorporate knowledge from experts and other key stakeholders from the beginning of the scoping review - Articulate rationale for expert consultation - Specify degree of involvement for expert consultants - Consider consultant participation throughout the scoping review - Essential, not optional

## Conclusion

During the initial planning phase for “Outcomes and outputs affiliated with children’s advocacy centers in the United States: A scoping review”, we strongly felt the need to integrate a team based, mixed methods approach. The existent body of literature on scoping review methodology and scoping reviews on scoping reviews informed our process, however the literature rarely focused on actionable steps to enhance teamwork or clearly articulated a means to move beyond separate analyses of numeric and thematic data. While many scholars indirectly refer to using mixed methods within scoping reviews, few articles clearly described the scoping review process as a mixed method approach. Like Daubt et al [6], we found the process of engaging in a team-based approach to be extremely helpful in gaining a deeper understanding of the key themes surrounding outcomes and outputs affiliated with CACs. We also found that designing a framework that collected quantitative and qualitative variables and created the space to compare the results from numerical and content analyses enriched our findings. In conclusion, this article contributes to the discourse towards advancing scoping review methodology by including enhancements to the scoping review methodology that emphasize a team-based, mixed method approach. Table 7 represents a synthesis of customizations that can be used to enhance a team-based, mixed approach to scoping methodology.

**Table 7:** From Arskey and O’Malley and Beyond: customizations to enhance a team-based, mixed approach to scoping methodology

**Arksey & O’Malley Framework Stage**	Recommended Enhancements*
**Stage 1:Specify the research question**	- Conduct a rapid review to scan the scope of the existent literature - Specify the population, concepts, and context - Clearly state the research question(s) and purpose - Use cues from the literature to assemble a research team with the appropriate knowledge and expertise - Identify authors with expertise in the field of interest - Keep the process iterative - Set a time line and communication plan with team members
**Stage 2:** **Identify relevant literature**	- Describe rationale for decisions that limit the scope, such as language or time frame - Invite a librarian to help guide the search - Seek diverse published and unpublished sources: internet, electronic databases, article reference lists, hand searching, networking at conferences, relevant professional organizations, dissertations, closed clinical trials, clinical practice guidelines, or the Grey Matters Checklist - Use a research, clinical practice, policy framework to identify the knowledge and expertise of team members - Identify and reach out to potential expert consultants - Create an a priori scoping review protocol - Consider using online management software such as REDCap - Train team members on how to use the online management software - Determine the variables of interest for data extraction per the research question - Discuss variable candidates with the research team - Develop a data charting framework: consider incorporating quantitative and qualitative components
**Stage 3:** **Select Studies**	- Clearly define inclusion and exclusion criteria for title and abstract search and full text search - Assign a unique identifying number to each article to be included in the full text review - Assign at least 2 team members to independently review titles and abstracts for inclusion - Specify the team member who will address any questions that arise with the selection of titles and abstracts - Assign 2 different team members to independently conduct a full text review for each article, and a third person to address questions or resolve discrepancies involving inclusion or exclusion - Consider assigning team members to review articles that match their knowledge and expertise - Integrate some aspect of interrater reliability assessment, such interrater agreement or a pooled kappa, during the study selection process - Use the Preferred Reporting Items for Systematic Reviews and Meta-Analyses Flowchart (PRISMA) to enhance reporting transparency
**Stage 4:** **Extracting, mapping, and charting the data**	- Pilot the data extraction process with at least 2 team members prior to launching it across the entire team - Data analysis: quantitative (numerical analysis), qualitative (specify qualitative method, such as content analysis) - Consider side by side comparison of quantitative and qualitative data
**Stage 5:** **Summarize, synthesize, and report the results**	- Present findings (charts, tables, maps, word clouds) - Relate the findings to the research question(s) and purpose - Discussion section should apply the findings within the context of current trends and future implications for research, practice, and policy
**Stage 6:** **Integrate expert consultation**	- Incorporate knowledge from experts and other key stakeholders from the beginning of the scoping review - Articulate rationale for expert consultation - Specify degree of involvement for expert consultants - Consider consultant participation throughout the scoping review - Essential, not optional

*Recommendations from this table/graphical abstract represent the collective efforts and scholarship of multiple scholars across the time frame of 2005 to 2020 including [1], [2],^4^,^6^,^7^,^11^,[13], [14], [15],^17^].

## Declaration of Competing Interests

X The authors declare that they have no known competing financial interests or personal relationships that could have appeared to influence the work reported in this paper.

## Appendix Supplementary materials

Image, application 1

Image, application 2

Image, application 3

Image, application 4

Image, application 5

Image, application 6

Image, application 7
