# Supplementary material for: From Arksey and O’Malley and Beyond: Customizations to enhance a team-based, mixed approach to scoping review methodology
Source: MethodsX. 2021 May 7;8:101375. doi: 10.1016/j.mex.2021.101375 (PMC8374523; doi:10.1016/j.mex.2021.101375)
Supplement: Supplementary file 1 [file mmc1.docx]

Supplementary Materials A. Disciplinary Backgrounds of First Authors

| Discipline of First Author | Frequency (N = 77) | Percent |
| --- | --- | --- |
| Psychology | 22 | 28.9% |
| Social Work | 17 | 22.4% |
| Nursing | 8 | 10.5% |
| Sociology | 5 | 6.6% |
| Child Abuse Medicine | 4 | 5.3% |
| Criminal Justice | 4 | 5.3% |
| National Children’s Alliance | 3 | 3.9% |
| Evaluation and Public Management | 2 | 2.6% |
| Law | 2 | 2.6% |
| Psychiatry | 2 | 2.6% |
| Other | 8 | 9.3% |
