# Supplementary material for: From Arksey and O’Malley and Beyond: Customizations to enhance a team-based, mixed approach to scoping review methodology
Source: MethodsX. 2021 May 7;8:101375. doi: 10.1016/j.mex.2021.101375 (PMC8374523; doi:10.1016/j.mex.2021.101375)
Supplement: Supplementary file 2 [file mmc2.pdf]

Outcomes affiliated with Child Advocacy Centers: A Scoping Review

PID 3280

Codebook

Data Dictionary Codebook

06/30/2020 12:48pm

Collapse all instruments Collapse all instruments

	#	Variable / Field Name	Field Label *Field Note*	Field Attributes (Field Type, Validation, Choices, Calculations, etc.)
Instrument: **Scoping Review: CACS** (scoping_review_cacs)				Collapse Collapse
	1	record_id	Record ID	text
	2	author	Author(s):	text
	3	title	Title of article:	text
	4	year	Year of publication:	text
	5	journal	Name of Journal:	text
	6	doi	DOI:	text
	7	link	Link to the article:	text
	8	article_pdf	Article:	file
	9	abstract	Abstract:	notes<br>Custom alignment: LV
	10	keyword	Keywords:	text
	11	auth_discipline	Discipline of first author:	text
	12	pdf	PDF	file
	13	reviewer	Reviewer:	dropdown<br>1 Zawacki<br>2 Westphaln<br>3 Vazquez-Westphaln<br>12 Ronis<br>4 Regoeczi<br>5 Panda<br>6 McDavid<br>7 Masotya<br>8 Lounsbury<br>9 Lee<br>10 Garg<br>11 QA
	14	exclude	Exclude after full text review:	radio<br>1 Yes<br>0 No<br>3 Don't know
	15	exclusion_reason	Reason for exclusion:	checkbox

		Show the field ONLY if:<br>[exclude] = '1'		1 | exclusion_reason___1 | Not in USA<br>2 | exclusion_reason___2 | Not in English<br>3 | exclusion_reason___3 | Abused individual is 18+ years old<br>4 | exclusion_reason___4 | Full text not available<br>5 | exclusion_reason___5 | Not R/T CAC<br>6 | exclusion_reason___6 | Other
	16	reason_other<br>Show the field ONLY if:<br>[exclusion_reason(6)] = '1'	Other:	text
	17	comments	Reviewer comments:	notes
	18	state<br>Show the field ONLY if:<br>[exclude] = '0'	Section Header: *SETTING OF PAPER*<br>US State (if noted):	dropdown<br>1 | Alabama - AL<br>2 | Alaska - AK<br>3 | Arizona - AZ<br>4 | Arkansas - AR<br>5 | California - CA<br>6 | Colorado - CO<br>7 | Connecticut - CT<br>8 | Delaware - DE<br>9 | Florida - FL<br>10 | Georgia - GA<br>11 | Hawaii - HI<br>12 | Idaho - ID<br>13 | Illinois - IL<br>14 | Indiana - IN<br>15 | Iowa - IA<br>16 | Kansas - KS<br>17 | Kentucky - KY<br>18 | Louisiana - LA<br>19 | Maine - ME<br>20 | Maryland - MD<br>21 | Massachusetts - MA<br>22 | Michigan - MI<br>23 | Minnesota - MN<br>24 | Mississippi - MS<br>25 | Missouri - MO<br>26 | Montana - MT<br>27 | Nebraska - NE<br>28 | Nevada - NV<br>29 | New Hampshire - NH<br>30 | New Jersey - NJ<br>31 | New Mexico - NM<br>32 | New York - NY<br>33 | North Carolina - NC<br>34 | North Dakota - ND<br>35 | Ohio - OH

				36 Oklahoma - OK<br>37 Oregon - OR<br>38 Pennsylvania - PA<br>39 Rhode Island - RI<br>40 South Carolina - SC<br>41 South Dakota - SD<br>42 Tennessee - TN<br>43 Texas - TX<br>44 Utah - UT<br>45 Vermont - VT<br>46 Virginia - VA<br>47 Washington - WA<br>48 West Virginia - WV<br>49 Wisconsin - WI<br>50 Wyoming - WY<br>51 Multiple States
	19	multiple_states<br>Show the field ONLY if:<br>[state] = '51'	Multiple States:	text
	20	community<br>Show the field ONLY if:<br>[exclude] = '0'	Residential Community:	radio<br>1 rural<br>2 urban<br>3 suburban<br>4 not specified<br>5 other
	21	community_other<br>Show the field ONLY if:<br>[community] = '5' and [exclude] = '0'	Other:	text
	22	purpose<br>Show the field ONLY if:<br>[exclude] = '0'	Section Header: *INTRODUCTION*<br>Study purpose:	notes
	23	aims<br>Show the field ONLY if:<br>[exclude] = '0'	Aim 1:<br>*if applicable*	notes
	24	aims_2<br>Show the field ONLY if:<br>[exclude] = '0' and [aims] <> ''	Aim 2:<br>*if applicable*	notes
	25	aims_3<br>Show the field ONLY if:<br>[exclude] = '0' and [aims_2] <> ''	Aim 3:<br>*if applicable*	notes
	26	aim_4<br>Show the field ONLY if:<br>[exclude] = '0' and [aims_3] <> ''	Aim 4:	notes
	27	aim_5<br>Show the field ONLY if:<br>[exclude] = '0' and [aim_4] <> ' '	Aim 5:	notes

	28	other_framework<br>Show the field ONLY if:<br>[exclude] = '0'	Does the study refer to a theory, framework, or model other than the CAC model?	yesno<br>1 Yes<br>0 No
	29	framework2<br>Show the field ONLY if:<br>[exclude] = '0' and [other_framework]='1'	Which theory, framework or model:<br>*one theory, framework, or model per box*	text
	30	framework3<br>Show the field ONLY if:<br>[exclude] = '0' and [other_framework]='1' and [framework2] <> ''	Additional theory, framework or model:<br>*one theory, framework, or model per box*	text
	31	framework4<br>Show the field ONLY if:<br>[exclude] = '0' and [other_framework]='1' and [framework3] <> ''	Additional theory, framework or model:<br>*one theory, framework, or model per box*	text
	32	framework5<br>Show the field ONLY if:<br>[exclude] = '0' and [other_framework]='1' and [framework4] <> ''	Additional theory, framework or model:<br>*one theory, framework, or model per box*	text
	33	framework6<br>Show the field ONLY if:<br>[exclude] = '0' and [other_framework]='1' and [framework5] <> ''	Additional theory, framework or model:<br>*one theory, framework, or model per box*	text
	34	framework7<br>Show the field ONLY if:<br>[exclude] = '0' and [other_framework]='1' and [framework6] <> ''	Additional theory, framework or model:<br>*one theory, framework, or model per box*	text
	35	framework8<br>Show the field ONLY if:<br>[exclude] = '0' and [other_framework]='1' and [framework7] <> ''	Additional theory, framework or model:<br>*one theory, framework, or model per box*	text
	36	framework9<br>Show the field ONLY if:<br>[exclude] = '0' and [other_framework]='1' and [framework8] <> ''	Additional theory, framework or model:<br>*one theory, framework, or model per box*	text
	37	framework10<br>Show the field ONLY if:<br>[exclude] = '0' and [other_framework]='1' and [framework9] <> ''	Additional theory, framework or model:<br>*one theory, framework, or model per box*	text
	38	branch<br>Show the field ONLY if:<br>[exclude] = '0'	Section Header: *POPULATION*<br>From the statement of purpose and aims of this paper, which branch of the CAC multidisciplinary team does this paper mainly involve?	dropdown<br>1 Medical team/services<br>2 Mental health team<br>3 Child protection services/ social work team<br>4 Law enforcement/police team<br>5 Prosecution/lawyers team (district attorney)<br>6 Victim/family advocacy team<br>7 Specific to forensic interview services

				8 CAC human resources (such as manager or directors)<br>9 combination
	39	pop_combination<br><br>Show the field ONLY if:<br>[exclude] = '0'	Combination:	text
	40	abuse_type<br><br>Show the field ONLY if:<br>[exclude] = '0'	What type of abuse is mentioned in this paper (choose all that apply)	checkbox<br>1 abuse_type___1 Physical<br>2 abuse_type___2 Sexual<br>3 abuse_type___3 Emotional<br>4 abuse_type___4 Neglect<br>5 abuse_type___5 Other
	41	other_abuse<br><br>Show the field ONLY if:<br>[abuse_type(5)] = '1'	other:	text
	42	race<br><br>Show the field ONLY if:<br>[exclude] = '0'	Is race/ethnicity of abused children and/or non-offending family members mentioned in this paper?	yesno<br>1 Yes<br>0 No
	43	race2<br><br>Show the field ONLY if:<br>[exclude] = '0' and [race]= '1'	Please select from the following, you may select more than one.	checkbox<br>1 race2___1 African American<br>2 race2___2 Asian American<br>3 race2___3 Pacific Islander<br>4 race2___4 Native American<br>5 race2___5 Caucasian<br>6 race2___6 Hispanic<br>7 race2___7 Other
	44	other_race<br><br>Show the field ONLY if:<br>[exclude] = '0' and [race2(7)]= '1'	other:	text
	45	design<br><br>Show the field ONLY if:<br>[exclude] = '0'	Section Header: *METHODS*<br>What is the primary design of the paper:	radio<br>1 Quantitative<br>2 Qualitative<br>3 Mixed approach<br>4 Concept or framework paper (not research)<br>5 Review of literature paper (not research)<br>6 Review of a guideline or a protocol<br>7 Systematic review<br>8 Metaanalysis<br>9 Scoping review<br>10 Integrative review<br>11 Case report/review<br>12 Narrative review<br>13 Doctoral dissertation<br>14 Masters thesis<br>15 Other
	46	design_other	Other:	text

		Show the field ONLY if: [exclude] = '0' and [design]='15'		
	47	quant_type<br><br>Show the field ONLY if: [design]= '1' and [exclude] = '0'	Quantitative design:	radio<br>1 Randomized Control Trial<br>2 Quasi-experiemental (not randomized trial)<br>3 Longitudinal (cohort study)<br>4 Cross-sectional study<br>5 Case control study<br>6 Other
	48	quant_other<br><br>Show the field ONLY if: [quant_type] = '6' and [exclude] = '0'	Other:	text
	49	variables<br><br>Show the field ONLY if: [design]= '1' and [exclude] = '0'	Study variables:	notes
	50	qual_type<br><br>Show the field ONLY if: [exclude] = '0'	Qualitative design:	radio<br>1 Narrative inquiry<br>2 Ethnography<br>3 Phenomenology<br>4 Action research<br>5 Other
	51	quanl_other<br><br>Show the field ONLY if: [qual_type] = '5' and [exclude] = '0'	Other:	text
	52	mixed_type<br><br>Show the field ONLY if: [exclude] = '0'	Mixed methods:	radio<br>1 Sequential explanatory (quant --> qual)<br>2 Sequential exploratory (qual --> quant)<br>3 Concurrent triangulation (integrated)<br>4 Other
	53	quanl_other_2<br><br>Show the field ONLY if: [mixed_type] = '4' and [exclude] = '0'	Other:	text
	54	impl_science<br><br>Show the field ONLY if: [exclude] = '0'	Does this paper refer to implementation science or implementation research?	radio<br>1 Yes<br>0 No<br>3 Don't know
	55	sample_size<br><br>Show the field ONLY if: [exclude] = '0'	Sample size:	text
	56	shared_data<br><br>Show the field ONLY if: [exclude] = '0'	Does this paper refer to any types of shared data sources, databases, or integrated data, linked data, or health information?	yesno<br>1 Yes<br>0 No
	57	shared_data2<br><br>Show the field ONLY if: "shared_data"='1' and [exclude] = '0'	Details about data:	notes

	58	outcomes_yn<br>Show the field ONLY if:<br>[exclude] = '0'	Section Header: *OUTCOMES (directly related to people)*<br>Does the paper address outcomes?	yesno<br>1 Yes<br>0 No
	59	outcome<br>Show the field ONLY if:<br>[outcomes_yn]='1' and [exclude] = '0'	Outcome 1:	notes
	60	operational_def<br>Show the field ONLY if:<br>[outcomes_yn]='1' and [exclude] = '0'	What is the operational definition/ How is this outcome measured?	notes
	61	subject<br>Show the field ONLY if:<br>[outcomes_yn]='1' and [exclude] = '0'	Who is the main subject of this outcome?	radio<br>1 Child<br>2 Family Member<br>3 Family unit<br>4 CAC Staff/MDT Member<br>5 Other
	62	outcome_type<br>Show the field ONLY if:<br>[outcomes_yn]='1' and [exclude] = '0'	Type of outcome:	radio<br>1 Economic<br>2 Academic<br>3 Policy<br>4 Physical Health<br>5 Mental Health<br>6 Other
	63	outcome_other<br>Show the field ONLY if:<br>[outcome_type]='6' and [exclude]='0'	Other:	text
	64	outcome2<br>Show the field ONLY if:<br>[outcomes_yn]='1' and [exclude] = '0' and [outcome] <> ''	Outcome 2:	notes
	65	operational_def2<br>Show the field ONLY if:<br>[outcomes_yn]='1' and [exclude] = '0' and [outcome2] <> ''	What is the operational definition/ How is this outcome measured?	notes
	66	subject2<br>Show the field ONLY if:<br>[outcomes_yn]='1' and [exclude] = '0' and [outcome2] <> ''	Who is the main subject of this outcome?	radio<br>1 Child<br>2 Family Member<br>3 Family unit<br>4 CAC Staff/MDT Member<br>5 Other
	67	outcome_type2<br>Show the field ONLY if:<br>[outcomes_yn]='1' and [exclude] = '0' and [outcome2] <> ''	Type of outcome:	radio<br>1 Economic<br>2 Academic<br>3 Policy<br>4 Physical Health<br>5 Mental Health<br>6 Other

	68	outcome_other2<br>Show the field ONLY if:<br>[outcome_type2]='6' and [exclude]='0'	Other:	text
	69	outcome3<br>Show the field ONLY if:<br>[outcomes_yn]='1' and [exclude] = '0' and [outcome2] <> ''	Outcome 3:	notes
	70	operational_def3<br>Show the field ONLY if:<br>[outcomes_yn]='1' and [exclude] = '0' and [outcome3] <> ''	What is the operational definition/ How is this outcome measured?	notes
	71	subject3<br>Show the field ONLY if:<br>[outcomes_yn]='1' and [exclude] = '0' and [outcome3] <> ''	Who is the main subject of this outcome?	radio<br>1 Child<br>2 Family Member<br>3 Family unit<br>4 CAC Staff/MDT Member<br>5 Other
	72	outcome_type3<br>Show the field ONLY if:<br>[outcomes_yn]='1' and [exclude] = '0' and [outcome3] <> ''	Type of outcome:	radio<br>1 Economic<br>2 Academic<br>3 Policy<br>4 Physical Health<br>5 Mental Health<br>6 Other
	73	outcome_other3<br>Show the field ONLY if:<br>[outcome_type3]='6' and [exclude]='0'	Other:	text
	74	outcome4<br>Show the field ONLY if:<br>[outcomes_yn]='1' and [exclude] = '0' and [outcome3] <> ''	Outcome 4:	notes
	75	operational_def4<br>Show the field ONLY if:<br>[outcomes_yn]='1' and [exclude] = '0' and [outcome4] <> ''	What is the operational definition/ How is this outcome measured?	notes
	76	subject4<br>Show the field ONLY if:<br>[outcomes_yn]='1' and [exclude] = '0' and [outcome4] <> ''	Who is the main subject of this outcome?	radio<br>1 Child<br>2 Family Member<br>3 Family unit<br>4 CAC Staff/MDT Member<br>5 Other
	77	outcome_type4<br>Show the field ONLY if:<br>[outcomes_yn]='1' and [exclude] = '0' and [outcome4] <> ''	Type of outcome:	radio<br>1 Economic<br>2 Academic<br>3 Policy<br>4 Physical Health<br>5 Mental Health<br>6 Other

	78	outcome_other4<br><br>Show the field ONLY if:<br>[outcome_type4]='6' and [exclude]='0'	Other:	text
	79	outcome5<br><br>Show the field ONLY if:<br>[outcomes_yn]='1' and [exclude] = '0' and [outcome4] <> ''	Outcome 5:	notes
	80	operational_def5<br><br>Show the field ONLY if:<br>[outcomes_yn]='1' and [exclude] = '0' and [outcome5] <> ''	What is the operational definition/ How is this outcome measured?	notes
	81	subject5<br><br>Show the field ONLY if:<br>[exclude] = '0' and [outcome5] <> ''	Who is the main subject of this outcome?	radio<br>1 Child<br>2 Family Member<br>3 Family unit<br>4 CAC Staff/MDT Member<br>5 Other
	82	outcome_type5<br><br>Show the field ONLY if:<br>[exclude] = '0' and [outcome5] <> ''	Type of outcome:	radio<br>1 Economic<br>2 Academic<br>3 Policy<br>4 Physical Health<br>5 Mental Health<br>6 Other
	83	outcome_other5<br><br>Show the field ONLY if:<br>[outcome_type5]='6' and [exclude]='0'	Other:	text
	84	outcome6<br><br>Show the field ONLY if:<br>[outcomes_yn]='1' and [exclude] = '0' and [outcome5] <> ''	Outcome 6:	notes
	85	operational_def6<br><br>Show the field ONLY if:<br>[outcomes_yn]='1' and [exclude] = '0' and [outcome6] <> ''	What is the operational definition/ How is this outcome measured?	notes
	86	subject6<br><br>Show the field ONLY if:<br>[outcomes_yn]='1' and [exclude] = '0' and [outcome5] <> ''	Who is the main subject of this outcome?	radio<br>1 Child<br>2 Family Member<br>3 Family unit<br>4 CAC Staff/MDT Member<br>5 Other
	87	outcome_type6<br><br>Show the field ONLY if:<br>[exclude] = '0' and [outcome6] <> ''	Type of outcome:	radio<br>1 Economic<br>2 Academic<br>3 Policy<br>4 Physical Health<br>5 Mental Health<br>6 Other

	88	outcome_other6<br>Show the field ONLY if:<br>[outcome_type6]='6' and [exclude]='0'	Other:	text
	89	outcome7<br>Show the field ONLY if:<br>[outcomes_yn]='1' and [exclude] = '0' and [outcome6] <> ''	Outcome 7:	notes
	90	operational_def7<br>Show the field ONLY if:<br>[outcomes_yn]='1' and [exclude] = '0'	What is the operational definition/ How is this outcome measured?	notes
	91	subject7<br>Show the field ONLY if:<br>[exclude] = '0' and [outcome7] <> ''	Who is the main subject of this outcome?	radio<br>1 Child<br>2 Family Member<br>3 Family unit<br>4 CAC Staff/MDT Member<br>5 Other
	92	outcome_type7<br>Show the field ONLY if:<br>[exclude] = '0' and [outcome7] <> ''	Type of outcome:	radio<br>1 Economic<br>2 Academic<br>3 Policy<br>4 Physical Health<br>5 Mental Health<br>6 Other
	93	outcome_other7<br>Show the field ONLY if:<br>[outcome_type7]='6' and [exclude]='0'	Other:	text
	94	outcome8<br>Show the field ONLY if:<br>[outcomes_yn]='1' and [exclude] = '0' and [outcome7] <> ''	Outcome 8:	notes
	95	operational_def8<br>Show the field ONLY if:<br>[outcomes_yn]='1' and [exclude] = '0' and [outcome8] <> ''	What is the operational definition/ How is this outcome measured?	notes
	96	subject8<br>Show the field ONLY if:<br>[exclude] = '0' and [outcome8] <> ''	Who is the main subject of this outcome?	radio<br>1 Child<br>2 Family Member<br>3 Family unit<br>4 CAC Staff/MDT Member<br>5 Other
	97	outcome_type8<br>Show the field ONLY if:<br>[exclude] = '0' and [outcome8] <> ''	Type of outcome:	radio<br>1 Economic<br>2 Academic<br>3 Policy<br>4 Physical Health<br>5 Mental Health<br>6 Other

	98	outcome_other8<br>Show the field ONLY if:<br>[outcome_type8]='6' and [exclude]='0'	Other:	text
	99	outcome9<br>Show the field ONLY if:<br>[outcomes_yn]='1' and [exclude] = '0' and [outcome8] <> ''	Outcome 9:	notes
	100	operational_def9<br>Show the field ONLY if:<br>[outcomes_yn]='1' and [exclude] = '0' and [outcome9] <> ''	What is the operational definition/ How is this outcome measured?	notes
	101	subject9<br>Show the field ONLY if:<br>[exclude] = '0' and [outcome9] <> ''	Who is the main subject of this outcome?	radio<br>1 Child<br>2 Family Member<br>3 Family unit<br>4 CAC Staff/MDT Member<br>5 Other
	102	outcome_type9<br>Show the field ONLY if:<br>[exclude] = '0' and [outcome9] <> ''	Type of outcome:	radio<br>1 Economic<br>2 Academic<br>3 Policy<br>4 Physical Health<br>5 Mental Health<br>6 Other
	103	outcome_other9<br>Show the field ONLY if:<br>[outcome_type9]='6' and [exclude]='0'	Other:	text
	104	outcome10<br>Show the field ONLY if:<br>[outcomes_yn]='1' and [exclude] = '0' and [outcome9] <> ''	Outcome 10:	notes
	105	operational_def10<br>Show the field ONLY if:<br>[outcomes_yn]='1' and [exclude] = '0' and [outcome10] <> ''	What is the operational definition/ How is this outcome measured?	notes
	106	subject10<br>Show the field ONLY if:<br>[exclude] = '0' and [outcome10] <> ''	Who is the main subject of this outcome?	radio<br>1 Child<br>2 Family Member<br>3 Family unit<br>4 CAC Staff/MDT Member<br>5 Other
	107	outcome_type10<br>Show the field ONLY if:<br>[exclude] = '0' and [outcome10] <> ''	Type of outcome:	radio<br>1 Economic<br>2 Academic<br>3 Policy<br>4 Physical Health<br>5 Mental Health<br>6 Other

	108	outcome_other10<br>Show the field ONLY if:<br>[outcome_type10]='6' and [exclude]='0'	Other:	text
	109	outcomes_imple_science<br>Show the field ONLY if:<br>[impl_science]='1' and [exclude] = '0'	Outcomes:	notes
	110	tot_outcomes<br>Show the field ONLY if:<br>[exclude] = '0'	How many total outcomes were addressed in this paper?	dropdown<br>1 | 1<br>2 | 2<br>3 | 3<br>4 | 4<br>5 | 5<br>6 | 6<br>7 | 7<br>8 | 8<br>9 | 9<br>10 | 10
	111	outputs_yn<br>Show the field ONLY if:<br>[exclude] = '0'	Section Header: *OUTPUTS (Directly related to the program)*<br>Does the paper address outputs?	yesno<br>1 | Yes<br>0 | No
	112	output<br>Show the field ONLY if:<br>[outputs_yn]='1' and [exclude] = '0'	Outputs 1:	notes
	113	op_def_output<br>Show the field ONLY if:<br>[outputs_yn]='1' and [exclude] = '0' and [output] <> ''	What is the operational definition/ How is this output measured?	notes
	114	output_address<br>Show the field ONLY if:<br>[exclude] = '0' and [output] <> ''	What service/program component does this output address?	dropdown<br>1 | Satisfaction<br>2 | Economic outputs<br>3 | Law enforcement<br>4 | Prosecution<br>5 | Forensic interview<br>6 | CPS/Social Work<br>7 | Victim and Family Advocates<br>8 | Medical services<br>9 | Mental health services<br>10 | Other
	115	output_address_other<br>Show the field ONLY if:<br>[exclude] = '0' and [output_address]='10'	Other:	text
	116	satis_type<br>Show the field ONLY if:<br>[output_address] = '1' and [exclude]='0' and [output] <> ''	Satisifaction type:	radio<br>1 | Caregiver<br>2 | Patient<br>3 | MDT staff

	117	output2<br><br>Show the field ONLY if:<br>[outputs_yn]='1' and [exclude] = '0' and [output] <> ''	Output 2:	notes
	118	op_def_output2<br><br>Show the field ONLY if:<br>[outputs_yn]='1' and [exclude] = '0' and [output2] <> ''	What is the operational definition/ How is this output measured?	notes
	119	output_address_2<br><br>Show the field ONLY if:<br>[exclude] = '0' and [output2] < > ''	What service/program component does this output address?	dropdown<br>1 Satisfaction<br>2 Economic outputs<br>3 Law enforcement<br>4 Prosecution<br>5 Forensic interview<br>6 CPS/Social Work<br>7 Victim and Family Advocates<br>8 Medical services<br>9 Mental health services<br>10 Other
	120	output_address_other_2<br><br>Show the field ONLY if:<br>[exclude] = '0' and [output_address_2]='10'	Other:	text
	121	satis_type_2<br><br>Show the field ONLY if:<br>[output_address] = '1' and [exclude]='0' and [output2] <> ''	Satisifaction type:	radio<br>1 Caregiver<br>2 Patient<br>3 MDT staff
	122	output3<br><br>Show the field ONLY if:<br>[outputs_yn]='1' and [exclude] = '0' and [output2] <> ''	Output 3:	notes
	123	op_def_output3<br><br>Show the field ONLY if:<br>[outputs_yn]='1' and [exclude] = '0' and [output3] <> ''	What is the operational definition/ How is this output measured?	notes
	124	output_address_3<br><br>Show the field ONLY if:<br>[exclude] = '0' and [output3] < > ''	What service/program component does this output address?	dropdown<br>1 Satisfaction<br>2 Economic outputs<br>3 Law enforcement<br>4 Prosecution<br>5 Forensic interview<br>6 CPS/Social Work<br>7 Victim and Family Advocates<br>8 Medical services<br>9 Mental health services<br>10 Other
	125	output_address_other_3<br><br>Show the field ONLY if:<br>[exclude] = '0' and [output_address_3]='10'	Other:	text

	126	satis_type_3<br>Show the field ONLY if:<br>[output_address] = '1' and [ex clude]='0' and [output3] <> ''	Satisifaction type:	radio<br>1 Caregiver<br>2 Patient<br>3 MDT staff
	127	output4<br>Show the field ONLY if:<br>[outputs_yn]='1' and [exclude] = '0' and [output3] <> ''	Output 4:	notes
	128	op_def_output4<br>Show the field ONLY if:<br>[outputs_yn]='1' and [exclude] = '0' and [output4] <> ''	What is the operational definition/ How is this output measured?	notes
	129	output_address_4<br>Show the field ONLY if:<br>[exclude] = '0' and [output4] < > ''	What service/program component does this output address?	dropdown<br>1 Satisfaction<br>2 Economic outputs<br>3 Law enforcement<br>4 Prosecution<br>5 Forensic interview<br>6 CPS/Social Work<br>7 Victim and Family Advocates<br>8 Medical services<br>9 Mental health services<br>10 Other
	130	output_address_other_4<br>Show the field ONLY if:<br>[exclude] = '0' and [output_ad dress_4]='10'	Other:	text
	131	satis_type_4<br>Show the field ONLY if:<br>[output_address] = '1' and [ex clude]='0' and [output4] <> ''	Satisifaction type:	radio<br>1 Caregiver<br>2 Patient<br>3 MDT staff
	132	output5<br>Show the field ONLY if:<br>[outputs_yn]='1' and [exclude] = '0' and [output4] <> ''	Output 5:	notes
	133	op_def_output5<br>Show the field ONLY if:<br>[outputs_yn]='1' and [exclude] = '0' and [output5] <> ''	What is the operational definition/ How is this output measured?	notes
	134	output_address_5<br>Show the field ONLY if:<br>[exclude] = '0' and [output5] < > ''	What service/program component does this output address?	dropdown<br>1 Satisfaction<br>2 Economic outputs<br>3 Law enforcement<br>4 Prosecution<br>5 Forensic interview<br>6 CPS/Social Work<br>7 Victim and Family Advocates<br>8 Medical services<br>9 Mental health services<br>10 Other

	135	output_address_other_5<br>Show the field ONLY if:<br>[exclude] = '0' and [output_address_5]='10'	Other:	text
	136	satis_type_5<br>Show the field ONLY if:<br>[output_address] = '1' and [exclude]='0' and [output5] <> ''	Satisifaction type:	radio<br>1 Caregiver<br>2 Patient<br>3 MDT staff
	137	output6<br>Show the field ONLY if:<br>[outputs_yn]='1' and [exclude] = '0' and [output5] <> ''	Output 6:	notes
	138	op_def_output6<br>Show the field ONLY if:<br>[outputs_yn]='1' and [exclude] = '0' and [output6] <> ''	What is the operational definition/ How is this output measured?	notes
	139	output_address_6<br>Show the field ONLY if:<br>[exclude] = '0' and [output6] <> ''	What service/program component does this output address?	dropdown<br>1 Satisfaction<br>2 Economic outputs<br>3 Law enforcement<br>4 Prosecution<br>5 Forensic interview<br>6 CPS/Social Work<br>7 Victim and Family Advocates<br>8 Medical services<br>9 Mental health services<br>10 Other
	140	output_address_other_6<br>Show the field ONLY if:<br>[exclude] = '0' and [output_address_6]='10'	Other:	text
	141	satis_type_6<br>Show the field ONLY if:<br>[output_address] = '1' and [exclude]='0' and [output6] <> ''	Satisifaction type:	radio<br>1 Caregiver<br>2 Patient<br>3 MDT staff
	142	output7<br>Show the field ONLY if:<br>[outputs_yn]='1' and [exclude] = '0' and [output6] <> ''	Output 7:	notes
	143	op_def_output7<br>Show the field ONLY if:<br>[outputs_yn]='1' and [exclude] = '0' and [output7] <> ''	What is the operational definition/ How is this output measured?	notes
	144	output_address_7<br>Show the field ONLY if:<br>[exclude] = '0' and [output7] <> ''	What service/program component does this output address?	dropdown<br>1 Satisfaction<br>2 Economic outputs<br>3 Law enforcement<br>4 Prosecution<br>5 Forensic interview<br>6 CPS/Social Work

				7 Victim and Family Advocates<br>8 Medical services<br>9 Mental health services<br>10 Other
	145	output_address_other_7<br>Show the field ONLY if:<br>[exclude] = '0' and [output_address_7]='10'	Other:	text
	146	satis_type_7<br>Show the field ONLY if:<br>[output_address] = '1' and [exclude]='0' and [output7] <> ''	Satisifaction type:	radio<br>1 Caregiver<br>2 Patient<br>3 MDT staff
	147	output8<br>Show the field ONLY if:<br>[outputs_yn]='1' and [exclude] = '0' and [output7] <> ''	Output 8:	notes
	148	op_def_output8<br>Show the field ONLY if:<br>[outputs_yn]='1' and [exclude] = '0' and [output8] <> ''	What is the operational definition/ How is this output measured?	notes
	149	output_address_8<br>Show the field ONLY if:<br>[exclude] = '0' and [output8] < > ''	What service/program component does this output address?	dropdown<br>1 Satisfaction<br>2 Economic outputs<br>3 Law enforcement<br>4 Prosecution<br>5 Forensic interview<br>6 CPS/Social Work<br>7 Victim and Family Advocates<br>8 Medical services<br>9 Mental health services<br>10 Other
	150	output_address_other_8<br>Show the field ONLY if:<br>[exclude] = '0' and [output_address_8]='10'	Other:	text
	151	satis_type_8<br>Show the field ONLY if:<br>[output_address] = '1' and [exclude]='0' and [output8] <> ''	Satisifaction type:	radio<br>1 Caregiver<br>2 Patient<br>3 MDT staff
	152	output9<br>Show the field ONLY if:<br>[outputs_yn]='1' and [exclude] = '0' and [output8] <> ''	Output 9:	notes
	153	op_def_output9<br>Show the field ONLY if:<br>[outputs_yn]='1' and [exclude] = '0' and [output9] <> ''	What is the operational definition/ How is this output measured?	notes
	154	output_address_9<br>Show the field ONLY if:<br>[exclude] = '0' and [output9] < > ''	What service/program component does this output address?	dropdown<br>1 Satisfaction<br>2 Economic outputs

				3 Law enforcement<br>4 Prosecution<br>5 Forensic interview<br>6 CPS/Social Work<br>7 Victim and Family Advocates<br>8 Medical services<br>9 Mental health services<br>10 Other
	155	output_address_other_9<br>Show the field ONLY if:<br>[exclude] = '0' and [output_address_9]='10'	Other:	text
	156	satis_type_9<br>Show the field ONLY if:<br>[output_address] = '1' and [exclude]='0' and [output9] <> ''	Satisifaction type:	radio<br>1 Caregiver<br>2 Patient<br>3 MDT staff
	157	output10<br>Show the field ONLY if:<br>[outputs_yn]='1' and [exclude] = '0' and [output9] <> ''	Outcome 10:	notes
	158	op_def_output10<br>Show the field ONLY if:<br>[outputs_yn]='1' and [exclude] = '0' and [output10] <> ''	What is the operational definition/ How is this output measured?	notes
	159	output_address_10<br>Show the field ONLY if:<br>[exclude] = '0' and [output10] <> ''	What service/program component does this output address?	dropdown<br>1 Satisfaction<br>2 Economic outputs<br>3 Law enforcement<br>4 Prosecution<br>5 Forensic interview<br>6 CPS/Social Work<br>7 Victim and Family Advocates<br>8 Medical services<br>9 Mental health services<br>10 Other
	160	output_address_other_10<br>Show the field ONLY if:<br>[exclude] = '0' and [output_address_10]='10'	Other:	text
	161	satis_type_10<br>Show the field ONLY if:<br>[output_address] = '1' and [exclude]='0' and [output10] <> ''	Satisifaction type:	radio<br>1 Caregiver<br>2 Patient<br>3 MDT staff
	162	tot_outputs<br>Show the field ONLY if:<br>[exclude] = '0'	How many total outputs were addressed in this paper?	dropdown<br>1 1<br>2 2<br>3 3<br>4 4<br>5 5<br>[illegible]

				6 6<br>7 7<br>8 8<br>9 9<br>10 10
	163	findings<br>Show the field ONLY if:<br>[exclude] = '0'	Section Header: *FINDINGS*<br>Findings:	notes<br>Custom alignment: LV
	164	cac_principle<br>Show the field ONLY if:<br>[exclude] = '0'	Which of the 10 CAC core principles best matches up with the themes presented in this paper (SELECT ONE)	dropdown<br>1 1, MDT<br>2 2, Cultural competency and diversity<br>3 3, Forensic interviews<br>4 4, Victim support & advocacy<br>5 5, Medical evaluation<br>6 6, Mental health<br>7 7, Case review<br>8 8, Case tracking<br>9 9, Organizational capacity (administrative policy & procedures)<br>10 10, Child-focused setting<br>11 Multiple (please provide more details below)
	165	multiple_details<br>Show the field ONLY if:<br>[cac_principle] = '11'	Multiple principles addressed:	notes
	166	limitations<br>Show the field ONLY if:<br>[exclude] = '0'	Section Header: *LIMITATIONS*<br>Limitations:	notes<br>Custom alignment: LV
	167	recommendation1<br>Show the field ONLY if:<br>[exclude] = '0'	Section Header: *FUTURE RECOMMENDATIONS*<br>Recommendation 1:	notes
	168	recommendation2<br>Show the field ONLY if:<br>[exclude] = '0' and [recommendation1] <>''	Recommendation 2:	notes
	169	recommendation3<br>Show the field ONLY if:<br>[exclude] = '0' and [recommendation2] <>''	Recommendation 3:	notes
	170	recommendation4<br>Show the field ONLY if:<br>[exclude] = '0' and [recommendation3] <>''	Recommendation 4:	notes
	171	recommendation5<br>Show the field ONLY if:<br>[exclude] = '0' and [recommendation4] <>''	Recommendation 5:	notes
	172	grant_funded<br>Show the field ONLY if:<br>[exclude] = '0'	Section Header: *FUNDING*<br>Grant funded/received financial compensation?	yesno<br>1 Yes<br>0 No
	173	funder	Funder:	text

		Show the field ONLY if:<br>[exclude] = '0'		
	174	scoping_review_cacs_complete	Section Header: *Form Status*<br>Complete?	dropdown<br>0 Incomplete<br>1 Unverified<br>2 Complete
