# Supplementary material for: From Arksey and O’Malley and Beyond: Customizations to enhance a team-based, mixed approach to scoping review methodology
Source: MethodsX. 2021 May 7;8:101375. doi: 10.1016/j.mex.2021.101375 (PMC8374523; doi:10.1016/j.mex.2021.101375)
Supplement: Supplementary file 3 [file mmc3.docx]

Supplementary Material C. Discrete outcomes as extracted from the articles (n = 31)

| Reference | Conceptual Definition | Operational Definition |
| --- | --- | --- |
| Wherry JN, Huey CC, Medford EA | Therapist ability to identify PTSD symptoms | Percent of PTSD symptoms correctly identified |
| Bonach, K & Heckert, A | Satisfaction with Organization | Defined as satisfaction with organization; measured through an adapted version of the Satisfaction with Organization Scale (SOS). Revised SOS contains five questions such as, "In most ways, this organization is close to my ideal" and uses a six point scale ranging from 1 (strongly disagree) to 6 (strongly agree). Scale was the 6-point response range for the SOS was reduced to a 5-point scale ranging from 1 (strongly disagree) to 5 (strongly agree) for consistency with other item responses in the survey. |
| Jones LM, Cross TP, Walsh WA, Simone M | Rates of anxiety and depression |  |
| Bounds DT, Edinburgh LD, Fogg LF, Saeywc, EM | Emotional distress | The Emotional Distress Scale is a self-reported measure of recent moods comprised of four items that assess sadness, stress/ pressure, discouragement/ hopelessness, or nervousness/feeling worried or upset in the past 30 days (Edinburgh, Harpin, Garcia, & Saewyc, 2013). Each item is scored from 0 to 4 with higher scores meaning higher distress. Internal consistency reliability for this sample has an a = 0.89, and factor analyses yields one factor that accounts for 67.8% of the variance in the score. |
| Wherry, JN and Dunlop, CE | Trauma symptoms in young children | TSCYC Trauma Symptom Checklist for Young Children |
| Walsh, WA, Cross, TP, & Jones, LM | Parent feelings of blame or doubt involving CSA | Maternal Self-report Support Questionnaire (MSSQ) was used to assess parental blame or doubt. The Blame and Doubt scale consists of eight items reflecting a parent's tendency to doubt a child's disclosure and question the role the child may have played in the sexual abuse. The eight items were (a) believed child about everything that happened, (b) could not help feeling angry with child, (c) questioned child's honesty about the abuse, (d) wondered what child might have done to stop the abuse from happening, (e) wondered if child could have stopped the abuse if she or he wanted to, (f) could not help resenting all the trouble child's disclosure caused, (g) wondered if child somehow brought the abuse on herself or himself, and (h) wondered if the abuse really happened to your child. |
| Smith, DW, Sawyer, GK, Jones, LM, Cross T, McCart, MR, and Ralston, ME | Maternal Support | The Maternal Self-report Support Questionnaire MSSQ was developed as a 40-item questionnaire assessing a mother's perceptions of her behavior in 3 theoretically informed domains of abuse-specific support: Belief in Child (degree of credence in child's abuse description), Emotional Support (empathy for child's distress, absence of rejecting or negative emotions), and Protective Action (keeping the child safe from further abuse). Mothers use a scale ranging from 0 (not at all like me) to 6 (very much like me) to indicate the degree to which items describe their attitudes and behavior since the discovery of CSA. |
| Edinburgh LD, Harpin, SB, Garcia, CM, & Saewyc, EM | Self-harm (cutting) behavior | Have you ever bruised, cut, or burned self? Never/Yes |
| Edinburgh L, Pape-Blabolil J, Harpin SB, Saewyc E | Physical findings, risk behaviors and trauma symptoms |  |
| Jones LM, Cross TP, Walsh WA, Simone M | Caregiver satisfaction with Investigation | Psychometric analyses of the Investigation Satisfaction Scale (ISS) identified two relatively independent components of caregivers' satisfaction with investigations: satisfaction with investigators' response to the abuse allegations (Investigator Response subscale) and satisfaction with the forensic interview experience (Interview Experience subscale). |
| Walsh WA, Cross TP, Jones LM, Simone M, Kolko DJ | Caregiver satisfaction | Responses were very satisfied, somewhat satisfied, somewhat unsatisfied, very unsatisfied |
| Bounds DT, Edinburgh LD, Fogg LF, Saeywc EM | Suicidal ideation, suicide attempts, and self-injury | Assessed through three questions that have been asked widely on school-based adolescent health surveys across the United States and Canada for more than 25 years: Have you ever hurt yourself on purpose (cutting, burns, bruises)? Have you ever seriously considered attempting suicide? Have you ever actually attempted suicide? |
| Wherry, J. N. and C. E. Dunlop | TSCC Trauma Symptom Checklist for Children | Children between the ages of 8 and 17 completed 268 TSCCs measures (ages 8-16) |
| Smith, DW, Sawyer, GK, Jones, LM, Cross T, McCart, MR, and Ralston, ME | Child general behavior and social maladjustment | The Child Behavior Checklist CBCL (Achenbach, 1991) is a widely used parent-report measure of general behavioral and social maladjustment designed for use with children ages 4-18 years. It consists of 20 social competence items and 118 items that are indicative of emotional and behavioral difficulties. The CBCL produces standardized scores for specific behavioral difficulties (e.g., Attention Problems) and broad-band T-scores for Internalizing Behavior, Externalizing Behavior, and Social Competence. These scores can be compared across race/ethnicity and gender. A wealth of data attests to the excellent psychometric properties of the CBCL (Achenbach, 1991). |
| Bonach, K & Heckert, A | Job Support | Job support is defined as perceived levels of social support with regard to their work as forensic interviewers. 3 dimensions of job support were assessed: support from outside the workplace, support from inside the workplace, and confiding variables. Operationalized using 11 job support items developed by Horwitx (2006). First factor included items regarding perceptions of support from professionals, colleagues, supervisors, and administrators and compliments about work and confiding at work, which reflected "internal job support." Second factor included perceptions of support from clients and the public, which reflected "external work related job support." Final factor included perceptions of support from family and friends and confiding outside of work, which reflected an "external social support" factor. Confiding outside of work is discouraged in the human services due to confidentiality ethical obligations, and standards of best practice; therefore, it makes sense that this item would not load satisfactorily with any of the job support factors. |
| Edinburgh LD, Harpin, SB, Garcia, CM, & Saewyc, EM | Suicide attempt | Have you ever tried to kill yourself? Never/Yes |
| Bounds DT, Edinburgh LD, Fogg LF, Saeywc EM | Exposure to traumatic events and PTSD screening | The UCLA PTSD-RI is a screening tool designed to assess exposure to traumatic events and PTSD symptoms. It can be used with children 7 years old and older: self-report version diagnosis for PTSD in the following areas: re-experiencing, avoidance/numbing, and arousal l. Psychometric properties and diagnostic criteria validity from the screen have been well-documented (Steinberg et al., 2004). With this sample, the Cronbach's a = 0.93 for the symptoms, and principal components analysis extracts 3 rotated components that correspond to re-experiencing, avoidance/numbing, and arousal. |
| Wherry, JN & Dunlop, CE | Overall trauma and sexually related symptomatology | Items for each of the screeners were determined based on their prediction of overall trauma and sexual-related symptomatology from the corresponding normative sample of the TSCC and TSCYC. For each screener, there are 12 items that comprise the General Trauma subscale including items that tap posttraumatic stress (PTS), anxiety, depression, dissociation, and anger; there are 8 items that comprise the Sexual Concerns subscale for each screener. The user of the measures does not have to be a licensed mental health practitioner but should be appropriately trained and supervised and able to apply cutoff scores for triage of children for either further assessment or treatment. |
| Smith, DW, Sawyer, GK, Jones, LM, Cross T, McCart, MR, and Ralston, ME | Social Desirability | The Marlowe-Crown Social Desirability Scale MCSDS measures the mother's general tendency to endorse socially acceptable response options. The scale consists of 33 true-false items on which participants rate their own personal attitudes and traits. Items are balanced between statements that are culturally acceptable but probably untrue, and statements that are probably true but are undesirable. The sum of socially desirable responses is tallied and used as a continuous measure of positive self-presentation. The MCSDS has shown high levels of both internal consistency and test-retest reliability (Crowne & Marlowe, 1964). |
| Edinburgh LD, Harpin, SB, Garcia, CM, & Saewyc, EM | Problem substance use | Problem substance abuse was a score created from a series of items asking about problems associated with drug or alcohol use, worded to allow for a cut-off score based on the DSM-IV diagnostic criteria; the scale was validated by Fulkerson, Harrison and Beebe (1999) using data from more than 70,000 youth participating in the 1995 Minnesota Student Survey. |
| Bonach, K & Heckert, A | Secondary Traumatic Stress | Used the secondary traumatic stress scale STSS, a scale that has 17 items with three subscales (intrusion, avoidance, arousal) designed to measures symptoms within the past 7 days associated with indirect exposure to traumatic events through the social worker's professional relationships with traumatized clients. STSS was modified in this study by asking respondents to indicate symptoms in the past 6-months because: (1) some forensic interviewers may not interview on a weekly basis and (2) typically there is a one-time exposure to the child/family trauma for forensic interviewer. |
| Smith,DW, Sawyer, GK, Jones, LM, Cross T, McCart, MR, and Ralston, ME | Perception of social support | My Family and Friends MFF measures the child's perception of general social support. Children rank members of their network to indicate to whom the child turns to first, second, and so on. Children then use a barometer-type scale (0 to 50) to indicate their level of satisfaction with support received from each network member. The interview takes an average of 30 minutes to complete, has good test-retest reliability, and has been effectively used with sexually abused children (Feiring, Taska, & Lewis, 1998). |
| Jensen, JM, Jacobson, M, Unrau, Y, & Robinson, RL | Child behavior and emotions | Parent rating of child behavior and emotions |
| Kellogg, ND | Trauma symptoms | Child psychosocial functioning (gathered from the child), reactions to abuse (child and parent), use of physical discipline (child and family), and the Trauma Symptom Checklist for Children, a reliable and valid instrument developed by Dr. John Briere. |
| Jensen, JM, Jacobson, M, Unrau, Y, & Robinson, RL | Parent Satisfaction at Pretest and Three-Month Follow-up |  |
| Wolfteich, P & Loggins, B | Re-victimization | Existence of any substantiated abuse report within 24 months of the index allegation. Re-referral is defined as a case with subsequent allegations within the 24 month period regardless of substantiation status. |
| Bonach, K, Mabry, JB, & Potts-Henry, C | Medical services satisfaction | If children were referred to a physician for medical evaluation, the physician was helpful. Participants chose from five responses ranging from strongly agree (4) to strongly disagree (1), with not applicable |
| Bonach, K, Mabry, JB, & Potts-Henry, C | Satisfaction with district attorney | Two survey items asking whether entity was courteous and helpful Participants chose from five responses ranging from strongly agree (4) to strongly disagree (1), with not applicable. |
| Bonach, K, Mabry, JB, & Potts-Henry, C | Satisfaction with victim advocacy entity | Two survey items asking whether entity was courteous and helpful Participants chose from five responses ranging from strongly agree (4) to strongly disagree (1), with not applicable |
| Bonach, K, Mabry, JB, & Potts-Henry, C | Non-offending satisfaction with law enforcement/police | 3 survey items related to the entity related to whether agency was courteous, helpful, and acting in a timely way. Participants chose from five responses ranging from strongly agree (4) to strongly disagree (1), with not applicable. |
| Bonach, K, Mabry, JB, & Potts-Henry, C | Satisfaction with MDT process | Series of 3 aggregate measures on mailed survey using likert scale: aggregate measure 1: coordination and information 1) I was given info to know what to expect at initial interview 2) I was given info to know what to expect after initial interview 3) child was questioned by too many professionals 4) forensic interview fit my schedule aggregate measure. 2: responsiveness and providing for clients' comfort 1) CAC agency personnel acted in timely way 2 )children were made to feel comfortable 3) I was made to feel comfortable aggregate measure. 3: Staff helpfulness 1) agency personnel were courteous 2) agency personnel were helpful. Participants chose from five responses ranging from strongly agree (4) to strongly disagree (1), with not applicable. |
