# Supplementary material for: From Arksey and O’Malley and Beyond: Customizations to enhance a team-based, mixed approach to scoping review methodology
Source: MethodsX. 2021 May 7;8:101375. doi: 10.1016/j.mex.2021.101375 (PMC8374523; doi:10.1016/j.mex.2021.101375)
Supplement: Supplementary file 4 [file mmc4.docx]

Supplementary Material D. Discrete outputs as extracted from the articles (n = 78)

| Reference | Conceptual Definition | Operational Definition |
| --- | --- | --- |
| Bracewell TE | Decision to prosecute | Accept or reject decision to prosecute CSA case |
| Thackeray JD, Scribano PV, & Rhoda, D | Familiarity with gold standard DV assessments | CAC Director is or is not familiar with the gold standards for DV assessment: The National Council of Juvenile and Family Court Judges' "Green Book" and the Family Violence Prevention Fund/Office for Victims of Crime's "Identifying and Responding to Domestic Violence: Consensus Recommendations for Child and Adolescent Health" |
| Miller A & Rubin D | Felony prosecution charges | The term "felony prosecution" is defined here as a prosecution which had one or more felony charges, regardless of whether the case also involved misdemeanor charges. Rates of felony prosecution were calculated as incident rates per 100,000 children in each district |
| Cross TP, Jones LM, Walsh WA, Simone M, Kolko D | Percentage of team interviews | We coded whether or not an interview was observed, and how many observers there were from each discipline (law enforcement, child protective services, etc.). We defined a team interview case as one in which professionals from at least two different disciplines observed. |
| Jackson SL | Child-friendly CAC facility | A child-friendly facility is intended to provide children and families with a safe, comfortable and neutral environment for conducting child interviews, as well as providing other important services. This comfortable environment is believed to contribute to children's accurate reports of an event. Developmental appropriateness: over half (52%) of both member and nonmember centers have waiting rooms and/or play areas that are geared towards younger children, with the remaining centers having waiting rooms and/or play areas that have activities available for all ages of children and adolescents. Based on CAC administrator perspectives/self-report. |
| Duron, JF | Decision to prosecute | Accept or reject decision to prosecute CSA case |
| Bracewell, TE | Case coordination | If both parties were present at both the initial staffing and the forensic interview the case was labeled as "full coordination." If both parties were present at either the forensic interview or the initial case staffing the case was labeled as "partial coordination." If both parties were not present at either the initial case staffing or the forensic interview the case was labeled as "no coordination." |
| Duron, JF and Cheung, M | Impact of repeated interviewing on forensic interviewer | Output is measured by analyzing retrospective qualitative debriefing data and used and unused film footage from 2000 and 2010 when two training videos were produced, along with current feedback via email from social work student actors about their memory of their experience. |
| Fisher, AK, Mackey, TD, Langendoen, C, Barnard, M | Forensic Interview findings | No findings, inconclusive, or consistent with sexual abuse. Interview outcomes had been determined following each interview by the forensic interviewer and clinical director working in conjunction to evaluate several factors provided by the child during the interview. The factors included the content, consistency, and feasibility of the report; the child's ability to provide peripheral details and sensory information; the affect and demeanor of the child during the interview; and the ability of the child to explain the report. |
| Williams, J, Nelson-Gardell, D, Faller, KC, Tishelman, A, Cordisco-Steele, L | Referral for an extended assessment | Child characteristics included 1) preschool children; 2) child maltreatment suspected without direct disclosure; 3) children who deny sexual abuse when other persuasive evidence exists; 4) children with developmental disabilities, 5) children with mental health problems; 6) cultural barriers to communication; 7) children who have made seemingly bizarre maltreatment allegations; 8) children with extensive child welfare histories; 9) children with inconsistent maltreatment allegations (e.g., recantation); 10) children with multiple types/suspected offenders/instances of maltreatment; 11) children who appear to have been highly traumatized by maltreatment; and 12) children who exhibit sexualized or sexual predatory behaviors. |
| Johnson, JL and AE Shelley | Prosecution case outcome | Questionnaire item: guilty or not guilty |
| Walsh, WA, Jones, LM, Swiecicki, CC | Timeliness of prosecution case review | Time between initial report and prosecution review |
| Young, TH, Williams, J, Nelson-Gardell, D | Functions of victim advocate role | Critical and non-critical duties:1. Greet and/or provide an orientation to children and families when they arrive at the CAC 2. Interview parents and caregivers to provide crisis intervention and/or support 3. Assessment of child/ family's attitudes about participation in the investigation/prosecutionUsing a Likert-like scale, participants rated the importance of each job duty according to the following: very important 4, important 3, moderate importance 2, little importance 1, and unimportant 0. Participants could also select the option "not my/advocate's job 5." |
| Pipe, M, Orbach, Y, Lamb, ME, Abbott CB, Steward, H | Filing of criminal charges | Suspect was arrested with charges filed |
| Hartley, DJ, Mullings, JL, Marquart | Decision to prosecute | Measured initially as (1) case accepted for prosecution; (2) case declined; (3) case diverted. Recoded to compare cases accepted for prosecution versus not accepted for prosecution |
| Carnes, CN, Wilson, C, Nelson-Gardell | Outcome of applying forensic evaluation model | 4 way classification of credible/non-credible and disclosure/non-disclosure or problematic disclosure |
| Meunier-Sham, J, Cross, TP, & Zuniga, L | Quality of SANE nurses examination | To what extent over time Pedi SANE nurses provided adequate information and interpretation for the QA physicians to be able to conduct a review. |
| Thraen, IM, Fraiser, L, Cochella, C, Yaffe, J, Goede, P | Usability of teleCAM for SA exams | Participants were given a set of written instructions with various tasks to complete: uploading images. The evaluator observed all of the TeleCAM interactions while taking notes. After the participants completed the tasks, they were given a follow-up survey which consisted of Likert type scale responses to 13 usability statements. |
| Walsh, WA, Lippert, T, Cross, TP, Maurice DM, Davison, KS | Indictment decision | The number of days between the law enforcement report and indictment date (i.e., when the grand jury made its decision that the case should be prosecuted or dropped). |
| Wolfteich, P and Loggins, B | Substantiation | A finding of "verified" or "some indicators" was documented with regard to the primary maltreatment allegation. |
| Joa, D & Edelson, MG | Charges filed | Whether the DA's office filed charges |
| Burton, DC, Stanley, D, Ireson, CL | Satisfaction with telemedicine consultations | Which supported clinical decision making in sexual abuse examinations, facilitated peer review of examinations and provided more timely feedback from the consultant on a 5-point scale. |
| Newman, BS & Dannenfelser, PL | Collaboration | No explicit definition was provided In quantitative data the options were separate but consulting informal joint approaching formal joint formal joint |
| Thackeray JD, Scribano PV, Rhoda D | Frequency of DV assessment | 1.What assessment method was used 2.Who did the assessment? 3.Who was present with the caregiver at the time of the assessment a "universal assesser" was defined as a CAC that assesses female caregivers for DV more than 75% of the time. |
| Miller A & Rubin D | Convictions for child sexual abuse | Prosecutions have four main outcomes: dismissal, pled guilty, found guilty in trial, and found not guilty in trial. The prosecution is deemed a "conviction" if the defendant pleads guilty before trial or is found guilty in trial of at least one of the charges; thus, the "conviction rate" in a given year is the number of prosecutions ending in conviction (pled guilty + found guilty in trial) divided by the total number of prosecutions. |
| Cross TP, Jones LM, Walsh WA, Simone M, Kolko D. | Case review | The percentage of CPS cases that had at least one interagency case review meeting. This variable was coded if multiple disciplines convened a scheduled case review meeting at least once in the days or weeks after intake. |
| Walsh, WA, Jones, LM, Swiecicki, CC | How quickly cases are resolved in the criminal justice system. | Time between prosecution review and criminal disposition outcome |
| Pipe, M, Orbach, Y, Lamb, ME, Abbott CB, Steward, H. | Case outcomes | Conviction, acquittal, dismissal of charges, final disposition (still active; diverted; all charges dismissed; found not guilty at trial; found guilty at trial; pled guilty; pled to reduced charges) |
| Carnes, CN, Wilson, C, Nelson-Gardell | Successful application of forensic evaluation model | Defined as any of the following outcomes: credible disclosure credible non-disclosure non-credible disclosure |
| Edelson, MG & Joa, D | Types of Sexual Abuse Charges and Numbers of Counts Charged for CAC and Non-CAC Samples Whose Cases Were Filed |  |
| Meunier-Sham, J, Cross, TP, & Zuniga, L | The crispness and clarity of MedScope images over time |  |
| Walsh, WA, Lippert, T, Cross, TP, Maurice DM, Davison, KS | Case resolution time | Number of days between the indictment date and case disposition date (i.e., plea or trial outcome). |
| Wolfteich, P and Loggins, B | Efficiency | The number of days from the initial abuse report until the formal substantiation status was determined by DCFS. |
| Joa, D & Edelson, MG | Verdict | Guilty or not guilty |
| Burton, DC, Stanley, D, Ireson, CL | Satisfaction | On a 5-point scale: very satisfied with the accessibility of educational conferences via telemedicine. |
| Newman, BS, Dannenfelser, PL, & Pendleton, D | Perceived degree of collaboration | (1) reasons for using the CAC; (2) examples of coordination facilitated by the center; and (3) ways the centers could be more helpful to them as investigators. |
| Thackeray JD, Scribano PV, Rhoda D | Barriers to DV assessment in the CAC setting | Defined by participant responses |
| Bracewell, TE | MDT impact on prosecution | Number of cases with decision to prosecute within cases that had more MDT members at meetings |
| Williams, J, Nelson-Gardell, D, Faller, KC, Tishelman, A, Cordisco-Steele, L | Barriers to performing extended assessment | Barriers included 1) lack of funding; 2) lack of availability of training, 3) community professionals lack training; and 4) key professionals do not support extended assessments. The number of barriers totaled for this variable resulted in scores ranging from zero to four. The mean for this measure was 2.27. |
| Walsh, WA, Jones, LM, Swiecicki, CC | How quickly cases move from initial report to criminal disposition outcome | Time between child abuse report and criminal disposition outcome |
| Meunier-Sham, J, Cross, TP, & Zuniga, L | Physician agreement with pedi SANE exam findings | The proportion of cases over time in which the reviewing physicians reported that they agreed with the Pedi SANE's evaluation of the examination findings. |
| Walsh, WA, Lippert, T, Cross, TP, Maurice DM, Davison, KS | Case processing time | Number of days between the law enforcement report and case disposition date. The total case processing time was the sum of the charging decision time and the case resolution time. |
| Wolfteich, P and Loggins, B | Arrest | Whether perpetrator was arrested |
| Joa, D & Edelson, MG | Sentence type | Prison vs. probation |
| Burton, DC, Stanley, D, Ireson, CL | Usefulness of the technology | Physicians rated the usefulness of the technology using a questionnaire with demonstrated validity and reliability. They rated nine statements about the usefulness of the colposcope and software, and 11 statements about the ease of use of the colposcope and the software, on a five-point scale (1 represented strongly agree and 5 represented strongly disagree). |
| Williams, J, Nelson-Gardell, D, Faller, KC, Tishelman, A, Cordisco-Steele, L | Total number of drawbacks to performing extended assessments | Drawbacks included 1) costliness; 2) concerns about programming and/or contamination; 3) proving of the case is more difficult in court; 4) trained professionals not available; and 5) inability to assure child safety. The number of drawbacks totaled for this variable resulted in scores ranging from zero to five. |
| Meunier-Sham, J, Cross, TP, & Zuniga, L | Physician agreement with Pedi SANE nurse treatment and follow up recommendations | The percentage of cases in which the reviewing physicians agreed with the Pedi SANE on treatment and follow-up recommendations. |
| Wolfteich, P and B Loggins | Prosecution | Whether charges were filed against the perpetrator |
| Joa, D & Edelson, MG | Sentence length | Length of sentence in months |
| Cross TP, Jones LM, Walsh WA, Simone M, Kolko D | Interview Observation Characteristics | Data was coded whether or not an interview was observed, and how many observers there were from each discipline (law enforcement, child protective services, etc.). The prototype was a case in which an interview specialist conducted the interview while representatives from at least two agencies watched. |
| Williams, J, Nelson-Gardell, D, Faller, KC, Tishelman, A, Cordisco-Steele, L | Total number of goals respective respondents thought were important for the extended assessment process | Goals were operationalized in the survey using a list of ten possible goals for extended assessments with responses rated on a Likert-like scale with 0 as not important and 4 as crucially important. For the purposes of this current analysis, the Likert-like scale was dichotomized with a "no" meaning not important, and responses 1 through 4 as important (thus, disregarding for this analysis the degree of importance accorded by respondents). Goals included 1) facilitating maltreatment disclosures; 2) assessing risk factors relevant to maltreatment other than child disclosure; 3) facilitating alleged offender prosecution; 4) gathering additional forensically relevant information; 5) assessing the general mental health of the child, including possible trauma; 6) assessing child risk factors (e.g., developmental, medical, psychosocial, and cultural) unrelated to maltreatment, which might impact the child's well-being; 7) assessing the child, family, and ecological/cultural strengths/protective factors; 8) making clinical recommendations related to the child or his/her family's needs; 9) providing information about the best interests of the child to the court in domestic disputes; and 10) providing information to the child protection agency about how to protect a child. The number of goals noted as "important" to any degree were totaled for a possible score of zero to ten. The mean for this measure was 9.54. |
| Young, TH, Williams, J, Nelson-Gardell, D | Documentation Characteristics | 1. Documenting your contacts with families and/or children 2. Entering information into a computer for purposes of case management/case tracking. "Using a Likert-like scale, participants rated the importance of each job duty according to the following: very important 4, important 3, moderate importance 2, little importance 1, and unimportant 0. Participants could also select the option "not my/advocate's job |
| Cross TP, Jones LM, Walsh WA, Simone M, Kolko D. | Joint law enforcement/child protective services investigation | This was coded when the police and child protective services investigators collaborated on the investigation. The collaboration included some combination of conducting investigative activities together, sharing information, and making decisions jointly. This could have occurred with or without a multidisciplinary team or team interview. To code this as "yes," the police and CPS investigators had to collaborate on forensic interviewing and planning investigative actions; communication about the case that fell short of this was coded "no." |
| Hlavka HR, Olinger SD, Lashley JL | Function and Value of Using Anatomical Dolls as a Demonstration Aid | Clothing details; sexual contact/nonsexual contact, including clarification of children's vocabulary for contact (e.g., "humping"); body positions; body-part details such as children's vocabulary for body parts; and penetration, specifically. |
| Hornor G | Legal outcomes | Filing with prosecutor, number of counts charged in filed cases, counts against biologic or step fathers who were alleged perpetrators, defendants pleading or being found guilty |
| Walsh WA, Cross TP, Jones LM, Simone M, Kolko DJ | Time from report to exam | Number of days |
| Anderson, GD, Anderson, JN, & Krippner, M | Use of orienting messages in FI | Orienting messages provide important information about the interview itself while preparing children for this unique style of interaction. Orienting messages used by interviewers were assigned to 1 of 7 categories: (1) Can't or won't say; (2) I don't know; (3) I don't understand; (4) correcting the interviewer; (5) asking a question or for clarification; (6) reality-based discussion; and (7) ignorant interviewer statements. |
| Vanderzee, KL, Pemberton, JR, Conners-Burrow, N, Kramer, T | Referral for counseling services | Documentation of referral for counseling services in AR-BEST (deidentified database covering all Arkansas CACs) |
| Williams, J, Nelson-Gardell, D, Faller, KC, Tishelman, A, Cordisco-Steele, L | Whether or not respondents had conducted extended assessments. | Using one survey question to which respondents could answer either "yes" or "no": "Have you actually conducted extended assessments?" An extended assessment, extended evaluation, or extended forensic evaluation [EFE] was defined for the purposes of this study as the planned and systematic process of conducting more than two interview sessions (typically 4- 6 sessions), with the same child and by the same interviewer/ evaluator, for the purpose of gathering information about allegations of child maltreatment |
| Anderson, GD, Anderson, JN, & Gilgun, JF | Interviewer use of narrative practice | During narrative event practice version children were asked to tell the interviewer "everything" or "all about" a specific, episodic, and preferably significant autobiographical event. Interviewers then followed up children's narratives with open-ended prompts intended to solicit further narrative. |
| Allen, B, Wilson, KL, & Armstrong, NE | Trauma focused CBT training | The structure for the TF-CBT training completed by Active clinicians was based on the Institute for Health care Improvement (2003) Breakthrough Series Learning Collaborative Model for the implementation of an evidence-based practice. The year-long endeavor consisted of a prework phase, three in-person training sessions, and two action/practice periods. As prework, clinicians were instructed to complete a Web-based training in TF-CBT (TF-CBTweb, www.musc.edu/tfcbt), read the treatment manual (Cohen, Mannarino, & Deblinger, 2006), and participate in a phone call review of the standardized trauma screening and assessment instrument used in the collaborative. Each of the inperson training sessions consisted of 2 days of skills-based training, including delivery of the treatment techniques, case conceptualization, engaging clients in trauma-focused treatment, and case consultation. The practice/action periods occurred between the in-person trainings and involved clinicians providing TF-CBT at their own agencies and attending monthly consultation calls with the trainer. These calls provided a structure for clinicians to present cases for feedback, discuss barriers, and ask questions regarding implementation and fidelity monitoring with the trainer and larger group. All training elements were completed with a trainer approved by the developers of TF-CBT to provide instruction in the model. |
| Allen, B & Johnson, JC | Clinician training and use of TF-CBT | Definition: TF-CBT, as defined in review of literature, is an empirically supported treatment (EST) that addresses both child and caregiver concerns resulting from the experience of trauma. TF-CBT is a component-based model that consists of individual sessions for the child, parallel sessions with the caregiver, and later conjoint caregiver-child sessions. Measured via self-report surveys that reflected who received training in TF-CBT and use of TF-CBT in clinical practice |
| Staudt, M and Williams-Hayes, M | Therapist attitudes toward and experiences with treatment manuals | These questions were adopted, with minor revisions, from the survey developed by Addis and Krasnow (2000). Specifically, the questions included: (a) Have you ever heard of treatment manuals? (b) How clear an idea do you have of what a treatment manual is? (c) How much thought have you given to the use of treatment manuals in practice? (d) How strong are your attitudes/feelings about the role of treatment manuals in practice? (e) How often do you use treatment manuals in your clinical work at the CAC? (f) How many treatment manuals do you use on a semiregular basis in your work at the CAC? (g) How would you describe your first experience with treatment manuals? |
| Lippert,T, Cross, TP, Jones, L, Walsh, W | Suspect confession in child sexual abuse cases | Data on suspects' confessions were drawn from case records at all sites using a data collection form developed for this project. Data abstracted from case records included documentation about suspect interviews/interrogations. Suspect interviews and interrogations are purportedly distinguished by the degree of accusation and confrontation involved, where interviews are described as intended to assess guilt or innocence and interrogations are intended to elicit confession once a suspect is believed to be guilty (Kassin & Gudjonsson, 2004). For the purposes of the current study, no distinction was made between interviews and interrogations because both permit an opportunity for confession. |
| Faller, KC and Nelson-Gardell, D | Case classification (credible disclosure/noncredible; disclosure/credible; nondisclosure/unclear) | Measured using the Disclosure Credibility Checklist. Interviewers check when there is a positive finding to the following categories - child made a verbal disclosure - child provided a demonstration of abuse - child provided a description of the abuse to someone else - child provided the majority of details from a first person perspective - child demonstrated freedom to correct interviewer - child demonstrated freedom to say "I don't remember," "I don't know," or "I don't understand" - disclosure was somewhat unstructured without rote quality - specific details recounted - disclosure is consistent with developmental level - emotional context - behavioral checklist results - corroborative information/confirmatory factors - motivation factors - alternative explanations |
| Lippert, T, Cross, TP, Jones, L, Walsh, W | Child disclosure of abuse (child denied, disclosed fully or partially, or recanted allegations of abuse for each forensic interview) | Children's full disclosures were defined as a disclosure over the course of any single forensic interview of all sexual activity (all known sexual acts, all known incidents) that came to be known throughout the investigation, whether by the child's own disclosure, the suspect's confession, or a witness's account. |
| Edinburgh, L, Saewyc, E, Levitt, C | Legal | Percentage of cases charged/prosecuted; percentage of charted cases with guilty verdict or plea |
| Lippert, T, Favre, T, Alexander, C, & Cross, TP | Delayed/declined mental health services | Families who did not start or declined therapy services for child despite referral for CSA |
| Hlavka HR, Olinger SD, Lashley JL | Consistency of disclosure when using anatomic dolls during FI | Consistency of verbal disclosure was measured in three situations: (a) young age of child, (b) limited verbal abilities of child, and (c) child had indicated a body part that he had previously verbalized. |
| Wherry JN, Huey CC, Medford EA | Referral and staffing practices | Percentage of clients referred to therapy and/or screening assessment. What percent of therapy and screening assessment referrals are made internally to paid staff members. |
| Brink, FW, Thackeray, JD, Bridge, JA, Letson, MM, & Scribano, PV | MDT decision regarding likelihood of CSA | Operational Definition: Each case evaluated for CSA by a MDT was rated by the MDT at the conclusion of the forensic interview and medical examination. Five point Likert rating scale to determine the likelihood of abuse was completed by MDT consensus. Although there are no known validated scales to assess likelihood of abuse, this scale was previously vetted by content experts within the various disciplines to establish content validity. For the purpose of this study, the likelihood of abuse was stratified into either high likelihood (score of 4 or 5) or low/indeterminate likelihood of CSA (score of 1, 2, or 3). Measured: Summarized by frequency. |
| Anderson, GD, Anderson, JN, & Gilgun, JF | Traditional practice version | In the traditional practice version, interviewers used fewer open-ended prompts by initially asking children to "tell about" some autobiographical topic or event but then placed less emphasis on soliciting further narrative and episodic memory by abandoning narrative prompts for more focused questioning. |
| Allen, B, Wilson, KL, & Armstrong, NE | Clinician beliefs regarding treatment of children experiencing trauma | The Child Trauma Clinical Beliefs Scale (CTCBS) was used to assess the degree to which clinicians' ascribe to traditional beliefs regarding the treatment of children experiencing trauma. Nondirective/Unstructured vs. Directive/Structured: Four items assesses the clinician's belief in the degree to which treatment sessions should be nondirective/unstructured or directive/structured. Higher scores reflect a more structured/directive approach, with lower scores suggesting a more unstructured/nondirective approach. Clinicians with a nondirective approach were significantly more likely to endorse a theoretical orientation traditionally ascribing to this belief (e.g., humanistic, psychodynamic) and clinicians with higher scores were more likely to endorse a more directive theoretical orientation (e.g., cognitive- behavioral). Children's Verbal Ability: Four items assessed the clinician's belief that children are capable of verbally describing their trauma experiences. Higher scores suggest a greater belief in children's ability to discuss traumatic events, and a lower score indicates a belief that children are unable to discuss such events. |
| Allen, B & Johnson, JC | Use of TF-CBT techniques in clinicians trained in TF-CBT | Definition: Survey listed 24 techniques that may commonly be used with children experiencing trauma. Of the 24 techniques were 5 components of TF-CBT, including provide psychoeducation about trauma, teach the caregiver behavioral child management skills, teach relaxation and other coping skills, cognitive restructuring techniques, and develop a narrative of the trauma. The TF-CBT core components of in vivo desensitization, conjoint parent- child sessions, and enhance personal safety were not included in the survey. Measured: Measured via surveys. Only those 103 clinicians who reported being both trained in and using TF-CBT on a regular basis were included in these analyses. |
| Faller, KC and Nelson-Gardell.D | Likelihood of sexual abuse | Categories; sexual abuse likely, sexual abuse unlikely, unclear - case was classified by the forensic interviewer based on their own conclusion about the likelihood of sexual abuse upon completing the 22 page data gathering form on each child |
| Edinburgh, L, Saewyc, E, Levitt, C | Physical evidence (genital findings, DNA evidence) among CAC cases only | Adams (2001) classification of sexual abuse |
| Lippert, T, Favre, T, Alexander, C, & Cross, TP | “Initiator” family type | Definition: Families who have received at least 1 therapy session for child following referral. |
| Smith, DW, Witte, TH, Fricker-Elhai, AE | Substantiated abuse allegations associated with CAC setting | Of all substantiated cases, those seen at the CAC were almost twice as likely to be referred for prosecution as those investigated by standard procedures |
| Hlavka HR, Olinger SD, Lashley JL | Child distancing during FI | Distancing includes a child's shift from his or her own body to the anatomical doll representing the child. |
| Wherry JN, Huey CC, Medford EA | Ability to identify evidence-based treatments | Percent of respondents correctly identifying evidence-based treatments. |
| Walsh WA, Cross TP, Jones LM, Simone M, Kolko DJ | Relationship of medical exams to filing criminal charges | Conducting follow-up case record reviews of prosecution and court records approximately 2 years after the case was enrolled in the research study |
| Brink, FW, Thackeray, JD, Bridge, JA, Letson, MM, & Scribano, PV | CPS Disposition outcome | Operational Definition: The CPS agency's disposition decision (substantiated, indicated, unsubstantiated) was similarly entered into the tracking system following completion of their investigation. CPS determined a report to be substantiated when the child disclosed and there was corroborative evidence; there was a credible witness supporting the allegation; and/or it involved other forms of confirmation deemed valid by the public CPS agency (professional judgment that the child has been abused or neglected). A report was indicated when CPS determined there was circumstantial, medical, or other isolated indicators of child abuse or neglect deemed valid by the public CPS agency but was lacking confirmation. A report was unsubstantiated when the investigation/assessment completed by the public CPS agency determined no occurrence of child abuse or neglect. Measured: Summarized by frequency. |
| Anderson, GD, Anderson, JN, & Gilgun, J | Supportive statements | Supportive statements included any word used by interviewers such as "mm hmm", "okay," or "uh-huh" expressed as a response to the child during the narrative building portion of the interview. Facilitators or supportive statements were offered by the interviewers throughout the child's narrative after the initial open-ended questions were posed to the child during rapport in both the traditional practice version and the narrative event practice version. |
| Allen, B, Wilson, KL, & Armstrong, NE | Clinicians' overall attitudes towards EBTs | The Evidence-Based Practice Attitudes Scale (EBPAS) was used to assess clinician's attitudes about various characteristics involved with implementation of EBPS. The scale asks about clinicians' beliefs that EBTs diverge from their typical practice, how appealing they find EBTs, openness to using new interventions, and the likelihood of using an intervention if required to do so. |
| Staudt, M and Williams-Hayes, M | Therapists' attitudes about the treatment manuals | 22 item questionnaire regarding attitudes about treatment manuals that were answered on a 5-point scale (1 = strongly disagree, 2 = disagree, 3 = neutral, 4 = agree, and 5 = strongly agree) and 9 items that assessed therapist knowledge about treatment manuals. These were answered on a 4-point scale (1 = not at all characteristic, 2 = somewhat characteristic, 3 = characteristic, and 4 = very characteristic). |
| Hlavka HR, Olinger SD, Lashley JL. | Child Communication during FI using anatomical dolls | Increased communication in response to the following situation(s): child's general tentativeness, unwillingness to provide details due to emotional affect, or the lack of language skills either because of very young age or lack of interview engagement |
| Wherry JN, Huey CC, Medford EA | Ability to identify reliable, valid, and normed assessments of abuse-related symptoms | Rating by respondents of a list of commonly administer general psychological tests as well as several reliable, valid, and normed tests. |
| Staudt, M and Williams-Hayes, M | Therapists’ attitudes toward EBP | Evidence based practice attitude scale (EBPAS) It consists of 15 items answered on a 5-point scale (1 = not at all, 2 = to a slight extent, 3 = to a moderate extent, 4 = to a great extent, and 5 = to a very great extent). Eight of the items measure therapist feelings about using new treatments or interventions, and 7 items ask therapists how likely it is they would adopt a new intervention if they received training in it, given certain conditions. The EBPAS contains 4 subscales: requirements (extent to which respondent would adopt a new practice if required by agency, supervisor, or state), appeal (extent to which respondent would adopt a new practice if it is intuitively appealing, makes sense, or is being used by colleagues), openness (extent to which respondent is open to trying new treatments), and divergence (extent to which respondent perceives research-based treatments as not clinically useful/less important than clinical experience). The EBPAS has good face, content, and construct validity and a Cronbach's alpha of .77 (Aarons, 2004). The Cronbach's alpha for the EBPAS in this study was .83, and for the subscales the alpha ranged from .68 for appeal to .98 for requirements (alpha for openness was .86 and for divergence it was .71). |
| Wherry JN, Huey CC, Medford EA | Identification of training needs | Rating of 5 different training priorities from 1 to 5. |
| Anderson, GD, Anderson, JN, & Gilgun, JF | Child disclosure of abuse | Disclosure was when a child made a verbal statement regarding a specific abuse allegation and the alleged perpetrator (e.g., "My dad touched me"). |
| Bracewell TE. | decision to prosecute | Accept or reject decision to prosecute CSA case |
| Thackeray JD, Scribano PV, Rhoda D | Familiarity with gold standard DV assessments | CAC Director is or is not familiar with the gold standards for DV assessment: The National Council of Juvenile and Family Court Judges' "Green Book" and the Family Violence Prevention Fund/Office for Victims of Crime's "Identifying and Responding to Domestic Violence: Consensus Recommendations for Child and Adolescent Health" |
| Miller A, Rubin D | Felony prosecution charges | The term "felony prosecution" is defined here as a prosecution which had one or more felony charges, regardless of whether the case also involved misdemeanor charges. Rates of felony prosecution were calculated as incident rates per 100,000 children in each district |
| Cross TP, Jones LM, Walsh WA, Simone M, Kolko D | Percentage of team interviews | We coded whether or not an interview was observed, and how many observers there were from each discipline (law enforcement, child protective services, etc.). We defined a team interview case as one in which professionals from at least two different disciplines observed. |
