# Supplementary material for: From Arksey and O’Malley and Beyond: Customizations to enhance a team-based, mixed approach to scoping review methodology
Source: MethodsX. 2021 May 7;8:101375. doi: 10.1016/j.mex.2021.101375 (PMC8374523; doi:10.1016/j.mex.2021.101375)
Supplement: Supplementary file 5 [file mmc5.docx]

Supplementary Material E. Interrater Proportion Agreement

| REDCap Article ID | Reviewer | Agreement | % agreement |
| --- | --- | --- | --- |
| 9 | BVW | 86 | 93.5% |
| 14 | SR | 79 | 85.9% |
| 73 | SR | 75 | 81.5% |
| 27 | KKW | 84 | 91.3% |
| 66 | KKW | 71 | 77.2% |
| 45 | WR | 83 | 90.2% |
| 81 | WR | 78 | 84.8% |
| 54 | KL | 82 | 89.1% |
| 28 | KL | 45 | 48.9% |
| 99 | KL | 78 | 84.8% |
| Total = 10 |  | 92 variables | Average 82.7% |
