# Supplementary material for: From Arksey and O’Malley and Beyond: Customizations to enhance a team-based, mixed approach to scoping review methodology
Source: MethodsX. 2021 May 7;8:101375. doi: 10.1016/j.mex.2021.101375 (PMC8374523; doi:10.1016/j.mex.2021.101375)
Supplement: Supplementary file 6 [file mmc6.docx]

Supplementary Material F. Outcome Thematic Clusters

CAREGIVER (11)

Satisfaction (8)

Satisfaction with investigation

General satisfaction with CAC services

Satisfaction with CAC services

Satisfaction with medical services

Satisfaction with district attorney

Satisfaction with victim advocacy entity

Satisfaction with law enforcement

Satisfaction with MDT process

Moms (3)

Feelings of blame or doubt

Maternal support

Social desirability

| CHILD (16)  Mental Health (13)  Anxiety  Depression  Emotional distress  Trauma symptoms in children  Self-cutting behavior  Risk behaviors  Overall trauma  Sexually related symptomatology  Problem substance use  Child perception of social support  Child emotions  Trauma Symptoms  Post-Traumatic Stress symptoms (PTSD)  Physical Health (3)  Physical findings from Forensic Medical Exam (FME)  Child Behavior  Re-victimization |
| --- |

| CAC STAFF (4)  Satisfaction (2)  Satisfaction with CAC organization  Satisfaction with telemedicine technology  Secondary Trauma (2)  Secondary traumatic stress  Impact of repeat CSA forensic interviews on interviewers |
| --- |
