# Supplementary material for: From Arksey and O’Malley and Beyond: Customizations to enhance a team-based, mixed approach to scoping review methodology
Source: MethodsX. 2021 May 7;8:101375. doi: 10.1016/j.mex.2021.101375 (PMC8374523; doi:10.1016/j.mex.2021.101375)
Supplement: Supplementary file 7 [file mmc7.docx]

Supplementary Materials G. Output Thematic Clusters

FORENSIC INTERVIEWS (FI) (16)

Forensic Interview Process (9)

Application of forensic evaluation model

Function and value of using anatomic dolls in a FI

Use of orienting messages in FI

Use of narrative practice in FI

Use of traditional practice in FI

Child distancing during FI

Use of supportive statements in fi

Child communication with using anatomic doll in FIs

Forensic interview observation status

Forensic Interview Findings (5)

FI findings (2)

Likelihood of sexual abuse

Consistency of disclosure

Credibility of disclosure

Forensic Interview Team (2)

Percentage of team FIs

Multidisciplinary FI

| PROSECUTION (22)  Decision to Prosecute (8)  Decision to prosecute (3)  Likelihood of prosecution  Filing of criminal charge (3)  Indictment decision  Number of counts charged  Court Results (7)  Convictions for child sexual abuse  Verdict (5)  Sentence length  Prosecution Timing (5)  Timeliness of prosecution  Case processing time (4)  Types of Charges (2)  Felony charges  Types of sexual abuse charges |
| --- |

FORENSIC MEDICAL EXAMS (10)

FME Process (3)

Quality of SANE examinations (2)

Usability to teleCAM for CSA FME

FME Findings (3)

Physical evidence (3)

Consultations (2)

Clinician satisfaction with resources

Usefulness of telehealth consultation

FME Timing (1)

Clinician belief regarding trauma symptoms (1)

| MENTAL HEALTH (10)  Therapists’ attitudes and beliefs (5)  Experiences with treatment manuals  Treatments for children experiencing trauma symptoms  Attitudes towards evidence-based treatments (EBT) (2)  Attitudes using treatment manuals  Therapists’ knowledge of EBTs (3)  Ability to identify EBTs (2)  Ability to recognize pediatric PTSD symptoms  Service Timing (2)  Delayed services  Declined services |
| --- |

| CASE COORDINATION (10)  Collaboration (4)  Collaboration (2)  Joint CPS and Law Enforcement  Documentation of collaboration  Referrals (4)  For extended assessment  For counseling services  Practices for making referrals  Families that received at least 1 referral  Case Review (2)  Amount of cases reviewed  MDT decision making |
| --- |

| DOMESTIC VIOLENCE (3)  Frequency of assessment for DV (1)  Gold standards for DV assessments (1)  Barriers to DV screening (1) |
| --- |

EXTENDED ASSESSMENTS (4)

Barriers (2)

Barriers to performing extended assessments (2)

Frequency (1)

Goals (1)

| TRAUMA FOCUSED COGNITIVE BEHAVIORAL THERAPY (4)  CBT training process (3)  CBT training needs (1) |
| --- |

LAW ENFORCEMENT (2)

Arrest (1)

Confession (1)

| CAC SETTING (1)  Child friendly setting (1) |
| --- |

| VICTIM ADVOCACY (1)  Function of victim advocates (1) |
| --- |
